# Memory CD8^+^ T cells mediate early pathogen-specific protection via localized delivery of chemokines and IFNγ to clusters of monocytes

**DOI:** 10.1126/sciadv.abf9975

**Published:** 2021-09-01

**Authors:** Marie Boutet, Zachary Benet, Erik Guillen, Caroline Koch, Saidi M’Homa Soudja, Fabien Delahaye, David Fooksman, Grégoire Lauvau

**Affiliations:** 1 Department of Microbiology and Immunology, Albert Einstein College of Medicine, 1301 Morris Park Avenue, Bronx, NY 10461, USA.; 2 Department of Pathology, Albert Einstein College of Medicine, 1300 Morris Park Avenue, Bronx, NY 10461, USA.; 3 Department of Genetics, Albert Einstein College of Medicine, 1301 Morris Park Avenue, Bronx, NY 10461, USA.; 4 Institut Pasteur de Lille, UMR1283/8199, 59000 Lille, France.

## Abstract

While cognate antigen drives clonal expansion of memory CD8^+^ T (CD8^+^ T_M_) cells to achieve sterilizing immunity in immunized hosts, not much is known on how cognate antigen contributes to early protection before clonal expansion occurs. Here, using distinct models of immunization, we establish that cognate antigen recognition by CD8^+^ T_M_ cells on dendritic cells initiates their rapid and coordinated production of a burst of CCL3, CCL4, and XCL1 chemokines under the transcriptional control of interferon (IFN) regulatory factor 4. Using intravital microscopy imaging, we reveal that CD8^+^ T_M_ cells undergo antigen-dependent arrest in splenic red pulp clusters of CCR2^+^Ly6C^+^ monocytes to which they deliver IFNγ and chemokines. IFNγ enables chemokine-induced microbicidal activities in monocytes for protection. Thus, rapid and effective CD8^+^ T_M_ cell responses require spatially and temporally coordinated events that quickly restrict microbial pathogen growth through the local delivery of activating chemokines to CCR2^+^Ly6C^+^ monocytes.

## Teaser

Protection in vaccinated hosts occurs quickly upon pathogen-specific memory CD8^+^ T cell–mediated activation of myeloid cells.

## INTRODUCTION

CD8^+^ T cells have the unique ability to sense and recognize antigens (Ags) derived from intracellular pathogens and tumors (*1*–*3*). Live attenuated vaccines using viral backbones [e.g., vaccinia and vesicular stomatitis virus (VSV)] or intracellular bacteria such as *Listeria monocytogenes* (*Lm*) and *mycobacteria* (Bacille Calmette-Guérin) are known to promote robust CD8^+^ T cell responses and establish a pool of systemic and tissue-resident long-lived memory CD8^+^ T (CD8^+^ T_M_) cells. These CD8^+^ T_M_ cells can rapidly react against immunizing Ags expressed in live vectors and provide immunity against life-threatening diseases (*4*–*6*). However, much investigation remains to be conducted to achieve a detailed understanding of (i) the role of cognate Ag and (ii) the sequences of events that need to take place for host protection.

It is well established that cognate Ag needs to be presented on dendritic cells (DCs) for optimal clonal reexpansion of both systemic and tissue-resident CD8^+^ T_M_ cells (*7*–*10*). Rapid clonal expansion ensures that sufficient numbers of pathogen-specific effector memory cells are generated to effectively sterilize an infection (*1*, *3*). We and others have shown in models of systemic bacterial and viral infections that both DCs and CCR2^+^Ly6C^+^ inflammatory monocytes also provide inflammatory signals that contribute to the early reactivation of CD8^+^ T_M_ cells in situ (*11*, *12*). Through the production of multiple inflammatory cytokines—i.e., interleukin-12 (IL-12), IL-18, IL-15, and type I interferon (IFN)—these cells can orchestrate rapid Ag-independent activation of CD8^+^ T_M_ cells (also known as “bystander” activation), including their differentiation into IFNγ-secreting natural killer group 2D (NKG2D^+^) effector cells (*11*, *13*–*16*). While this early cytokine-driven activation of CD8^+^ T_M_ cells contributes to innate protection, cognate Ag recognition is nevertheless required to achieve high levels of microbial pathogen-specific immunity, before clonal expansion occurs. Several mechanisms are likely to account for the rapid Ag-dependent CD8^+^ T_M_ cell–mediated protection, which include not only direct cytolysis of infected cells but also secretion of cytokines [IFNγ and tumor necrosis factor–α (TNFα)] and chemokines (CCL3) (*1*, *17*, *18*). However, how exactly cognate Ag versus inflammation programs CD8^+^ T_M_ cells during reactivation is unknown, and no studies to date have provided a comprehensive picture of these processes. The early transcriptional gene expression and effector program that is specifically triggered in CD8^+^ T_M_ cells upon early cognate Ag recognition is not known. This information is essential to further understand how cognate Ag enables CD8^+^ T_M_ cells to achieve immunized host protection early on and mediate the rapid control of pathogen growth and spreading in situ.

IFNγ is known to be an essential effector cytokine produced by activated effector CD8^+^ (and CD4^+^) T cells, which has complex and pleiotropic effects on immune cells (*19*). These include, for example, favoring T helper 1 cell and M1-type macrophage differentiation, promoting Ag presentation, and the production of microbicidal molecules. In this context, we have shown that IFNγ signaling to CCR2^+^Ly6C^+^ monocytes and, to some extent, neutrophils is key to induce them to produce effector molecules such as TNFα and CXCL9 (*20*). TNFα is absolutely required for immunized host protection during a recall *Lm* infection (*17*, *21*–*23*) through the potent induction of reactive oxygen species (ROS) by both CCR2^+^Ly6C^+^ monocytes and neutrophils (*17*). However, whether IFNγ signals are sufficient, or other signals are needed in conjunction, for effective protection to take place is also not known. In several models of infection including *Lm*, IFNγ is produced independently from cognate Ag (*11*, *12*, *14*), which further underscores the need to understand how cognate Ag may potentiate or contribute to IFNγ-mediated protection.

One notion illustrated across multiple studies and that accounts for how quick CD8^+^ T_M_ cells can protect immunized hosts is that these cells enable the rapid containment and effective elimination of microbial pathogens at portals of entry (*24*, *25*). CD8^+^ T_M_ cells can rapidly traffic to the sites of infection via chemotaxis (e.g., CXCR3 and CCR5) and adhesion [lymphocyte function-associated antigen 1 (LFA-1) and loss of L-selectin]. Proof-of-concept studies have used models of systemic viral (vaccinia, VSV, and lymphocytic choriomeningitis virus) and bacterial (*Lm*) infections in which microbial pathogens are rapidly captured in subcapsular draining lymph nodes (dLNs) or splenic marginal zone CD169^+^ macrophages and drive subsequent homing of CD8^+^ T_M_ cells in response to chemotactic cues (e.g., CXCL9 and CXCL10) produced by innate immune and structural cells (*24*–*27*). The massive Ag-independent recruitment of memory cells also leads to inflammation-driven activation of Ag-irrelevant CD8^+^ T_M_ cells (*26*). While comparable chemotactic mechanisms are also documented in the case of CD8^+^ and CD4^+^ tissue-resident T (T_RM_) cells in models of skin and vaginal viral infections, initiation of the rapid mucosal immune response by T_RM_ cells is largely dependent on initial cognate Ag recognition, leading to the establishment of a rapid antiviral state that restricts pathogen spreading (*28*–*31*). It is likely that initial chemotactic cues from tissue-resident cells involved in microbial pathogen capture (DCs and macrophages) enable CD8^+^ T_M_ cell recruitment to infectious foci and that the memory cells then help to quickly amplify and guide the recruitment of more immune effector cells through the secretion of both Ag-dependent and Ag-independent cytokines and chemokines. However, the exact sequences of events and whether memory cells represent the major orchestrators of the rapid amplification of the immune response and associated host protection are not known.

In the current work, we provided a comprehensive analysis of the cellular and molecular mechanisms by which cognate Ag programs and orchestrates early CD8^+^ T_M_ cell–mediated pathogen-specific protection in vaccinated hosts undergoing a recall infection. We reveal the cognate Ag–driven transcriptome of reactivated CD8^+^ T_M_ cells and precisely dissect the link between cognate Ag stimulation and CD8^+^ T_M_ cell–derived IFNγ production, a major protective cytokine produced independently from cognate Ag. Our results show that cognate Ag on DCs mediates CD8^+^ T_M_ cell arrest in infection foci where blood-derived CCR2^+^Ly6C^+^ monocytes have accumulated via CD8^+^ T_M_ cell–independent chemotactic cues. Here, CD8^+^ T_M_ cells deliver localized IFNγ and a set of cognate Ag–triggered chemokines, CCL3, CCL4, and XCL1. We also reveal that IFNγ signals, while necessary to drive full CCR2^+^Ly6C^+^ monocyte activation (TNFα and CXCL9), are not sufficient. We found that IFNγ signals are required for CCR2^+^Ly6C^+^ monocytes to become responsive to chemokine signals that drive their activation and license them with highly effective microbicidal functions for rapid pathogen containment and killing.

## RESULTS

### Cognate Ag versus inflammation triggers a broad range of functional pathways in CD8^+^ T_M_ cells

**Fig. 1.**
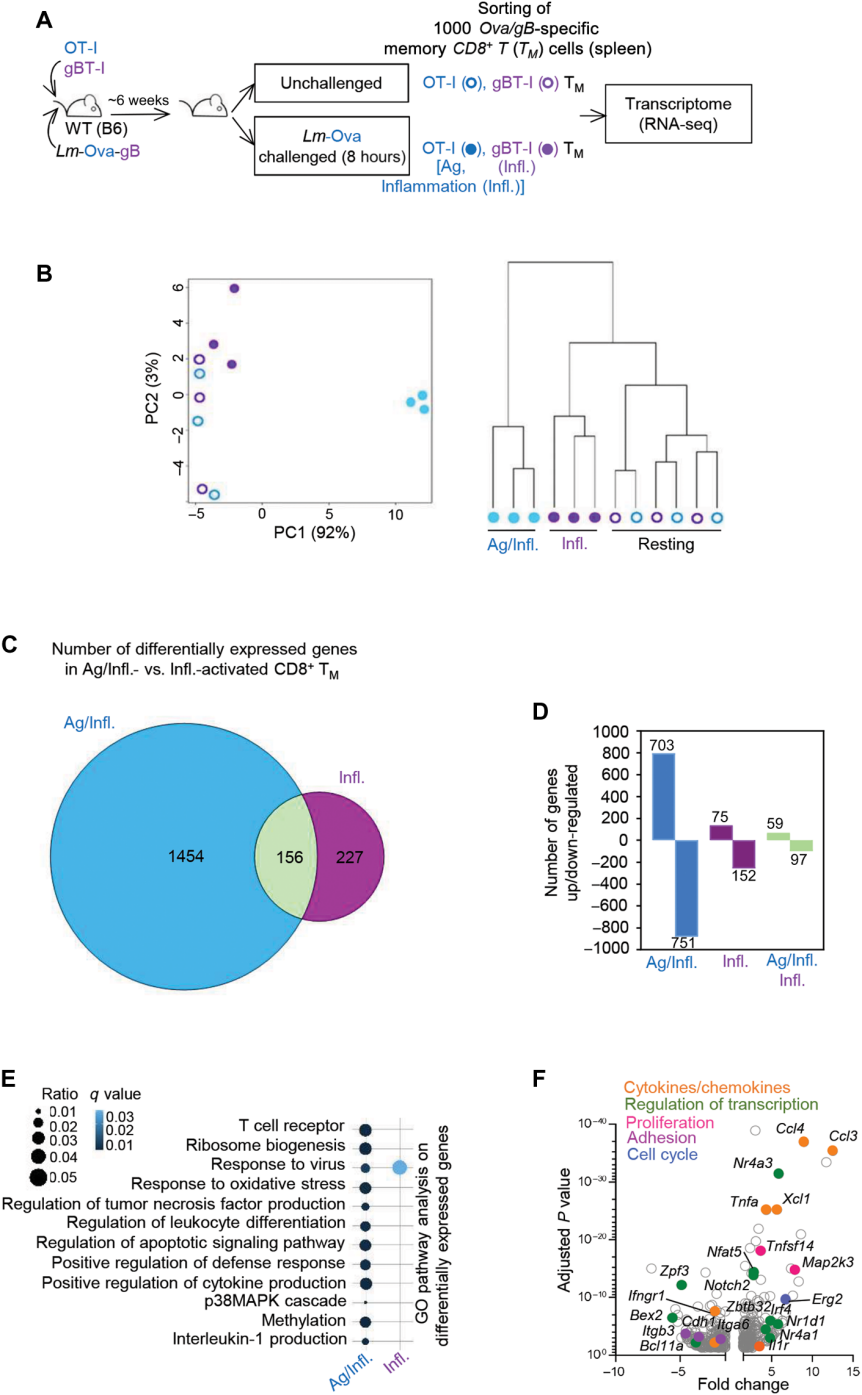
Transcriptomic profiling of Ag/inflammation-activated versus inflammation-activated CD8^+^ T_M_ cells. (**A**) Schematic of experimental design. Age-matched WT B6 female mice (CD45.2^+/+^) grafted with tomato-expressing (Td^+^) OT-I and CD45.1^+/+^ gBT-I cells were immunized with 10^4^ *Lm*-Ova-gB and ~6 weeks later challenged or not with 10^6^ *Lm*-Ova. After 8 hours, 1000 OT-I T_M_ (Ag/Infl., blue) and gBT-I T_M_ (Infl., purple) were flow-sorted from harvested mouse spleens based on CD8, CD3, Tomato (Td^+^), and CD45.1 expression, and samples were prepared for RNA sequencing (RNA-seq) analysis. (**B**) PCA plot (left) and clustering tree (right) of Ag/Infl. (OT-I)–stimulated versus Infl. (gBT-I)–stimulated T_M_ cells at steady state and after challenge. Each dot represents an individual mouse, and the number in parentheses indicates the percent of variance. Each set of samples (OT-I and gBT-I) was processed in three biologically independent replicate experiment. (**C**) Venn diagrams comparing the numbers of differentially expressed genes Ag/Infl. (OT-I)–stimulated versus Infl. (gBT-I)–stimulated T_M_ cells from secondary challenged mice (fold change, ±1.5; adjusted *P* < 0.05). The number of overlapping genes is specified in the green circle. (**D**) Bar graphs representing the number of genes up- and down-regulated from the Venn diagram analysis. (**E**) Representation of the top GO pathway analysis between Ag/Infl.-activated versus Infl-activated T_M_ cells. The size and color of dots are proportional to the number of genes under a specific term and the adjusted *P* value, respectively. MAPK, mitogen-activated protein kinase. (**F**) Volcano plot representing the significantly up- and down-regulated genes in Ag/Infl-activated (OT-I) T_M_ cells 8 hours after the recall challenge infection.

To understand how cognate Ag orchestrates CD8^+^ T_M_ cell early reactivation and programming in situ, we conducted a genome-wide transcriptional analysis of pathogen-specific memory cells undergoing reactivation in the presence or absence of their cognate Ag (Fig. 1). Naïve Ova_257–264_/K^b^-specific OT-I and gB_498–505_/K^b^-specific gBT-I T cell receptor (TCR) transgenic T cells were adoptively transferred to wild-type (WT) C57BL/6 (B6) mice that were immunized the next day with *Lm* expressing both T cell epitopes (*Lm*-Ova-gB). Six weeks later, immunized mice were challenged with *Lm* expressing Ova only (*Lm*-Ova), and we monitored OT-I and gBT-I T_M_ cell activation (Fig. 1A). This experimental setup enabled us to track memory cells that either “see” (OT-I, Ag/inflammation (Infl.)-activated) or do not see (gBT-I, Infl.-activated) their cognate Ag. The memory cells were flow-sorted from 8-hour-challenged or control unchallenged mice and subjected to transcriptomic analysis (Fig. 1B). Two-dimensional principal components analysis (PCA) (Fig. 1B, left) and hierarchical clustering (Fig. 1B, right) revealed that OT-I T_M_ cells (Ag/Infl.-activated) clustered separately from gBT-I (Infl.-activated) and resting T_M_ (unchallenged) cells that grouped close together. Thus, cognate Ag triggering drives a significantly distinct transcriptional profile in the CD8^+^ T_M_ cells. A total of 1837 genes were differentially expressed (*P* < 0.05; fold change, >1.5) in activated (Ag/Infl. + Infl.) versus resting T_M_ cells, with the vast majority (1454, i.e., ~79%) driven by Ag stimulation only and a smaller proportion triggered by inflammatory signals only (227, i.e., ~12%) (Fig. 1C and table S1). Only 156 genes (i.e., ~9%) among the differentially expressed genes were common between Ag-activated and inflammation-activated CD8^+^ T_M_ cells. While Ag stimulation induced similar numbers of up- and down-regulated genes, respectively 703 and 751, inflammation favored the expression of a higher proportion of down-regulated genes (152 versus 75 genes) including genes involved in cell adhesion and migration (*Cd44*, *Cd27*, *Itgax*, and *S1pr5*; Fig. 1D and table S1). Common genes were more similarly distributed between up-regulation and down-regulation. Further analysis of the genes differentially expressed in Ag-stimulated versus inflammation-stimulated CD8^+^ T_M_ cells using biological process gene ontology (BP-GO) pathway analysis revealed that cognate Ag, but not inflammation, promoted a wide range of biological functions related to TCR signaling, leukocyte differentiation, apoptosis, and cytokine expression (Fig. 1E and table S2). To achieve deeper understanding into the molecular mechanisms by which Ag stimulation modulates the early programming of CD8^+^ T_M_ cells, we plotted the fold change over respective adjusted *P* values of all differentially expressed genes (Fig. 1F). The most highly expressed genes in Ag-activated CD8^+^ T_M_ cells encoded for chemokines and cytokines (*Ccl4*, *Ccl3*, *Xcl1*, and *Tnfa*), important transcriptional regulators (*Nr4a3*, *Nr4a1*, *Nfat5*, *Zbtb32*, and *Irf4*), and proteins involved in proliferation/survival (*Tnfsf14* and *Map2k3*) and cell cycle (*Erg2*). Notably, expression of genes encoding adhesion molecules was largely down-regulated (*Itgb6*, *Itgb3*, and *Cdh1*) (Fig. 1F). In summary, cognate Ag stimulation endows CD8^+^ T_M_ cells with a robust early multifunctional gene expression program, among which the most significantly up-regulated genes encode for chemokines and a specific set of transcription factors.

### Memory CD8^+^ T cells produce an early and coordinated burst of chemokines upon cognate Ag recognition

**Fig. 2.**
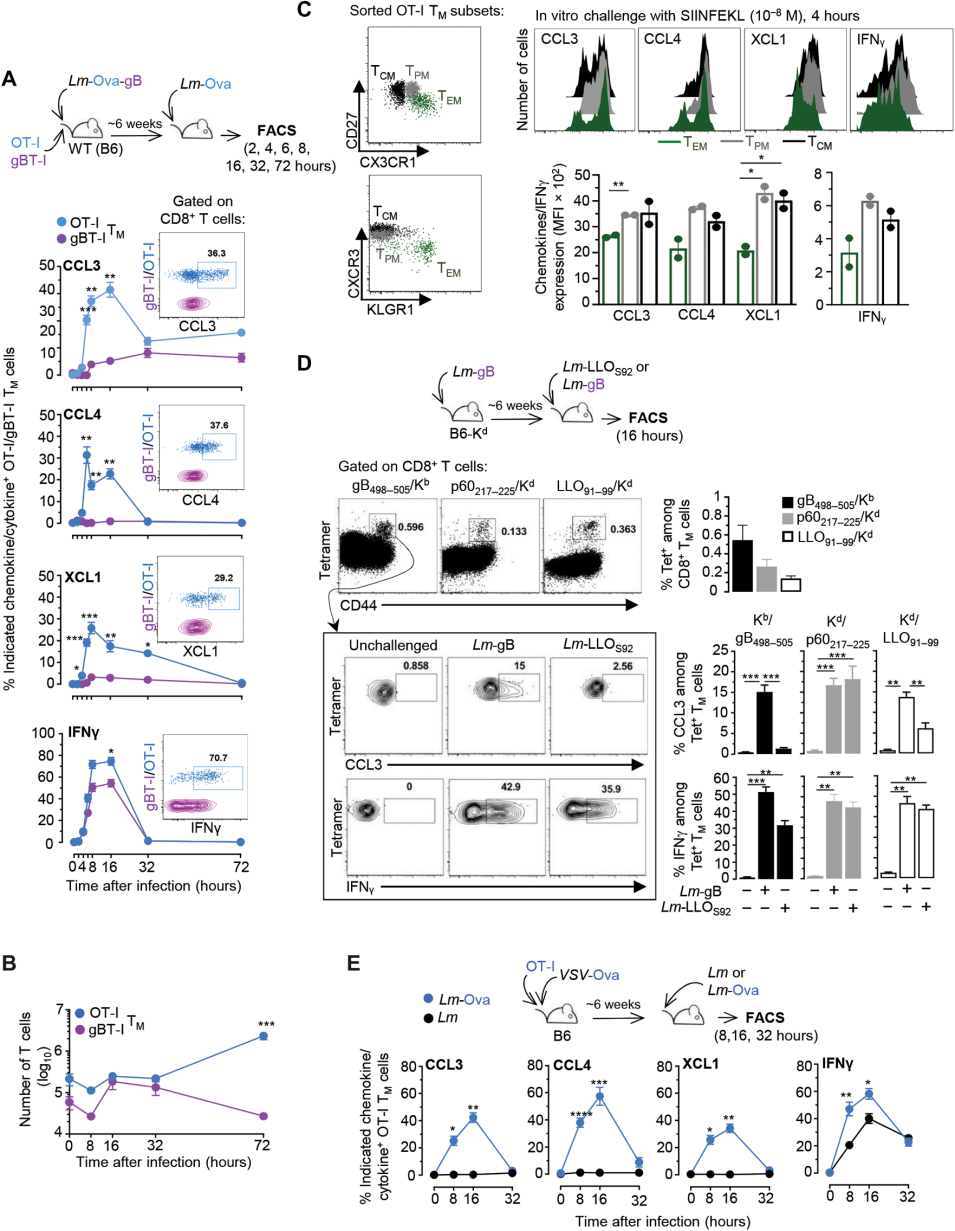
Cognate Ag recognition triggers the rapid and coordinated production of chemokines by CD8^+^ T_M_ cells. (**A** to **C**) WT mice transferred with OT-I Td^+^ and CD45.1^+/+^ gBT-I cells were immunized with 10^4^ *Lm*-Ova-gB and challenged ~6 weeks later with 10^6^ *Lm*-Ova for 2, 4, 6, 8, 16, 32, and 72 hours. At these times, spleen cells were incubated with GolgiPlug/Stop for 4 hours and stained for cell surface CD8, CD3, CD45.1, and intracellular CCL3, CCL4, XCL1, and IFNγ. (A) Kinetics of chemokines and IFNγ accumulation in OT-I (blue) and gBT-I (purple) T_M_ cells and representative overlaid dot plots of the staining. (B) Number of OT-I and gBT-I T_M_ cells at indicated times after recall infection. (C) OT-I T_M_ cell subsets flow-sorted from the spleens of OT-I–transferred *Lm*-Ova–immunized mice, based on CX3CR1 and CD27 expression (T_EM_, CX3CR1^hi^CD27^low^; T_PM_, CX3CR1^int^CD27^hi^; T_CM_, CX3CR1^low^CD27^hi^). OT-I T_M_ cell subsets were next stimulated for 4 hours with SIINFEKL peptide (10^−8^ M) before staining. Fluorescence-activated cell sorting (FACS) histograms and graphs show expression of indicated chemokine^+^ and IFNγ^+^ OT-I T_M_ cell subsets (*n* = 2 mice). (**D**) B6-K^d^ mice immunized with *Lm*-gB were challenged ~6 weeks later with *Lm*-gB or *Lm*-LLO_S92_ for 16 hours. Polyclonal CD8^+^ T_M_ cells were monitored using indicated Tet. The frequency of Tet^+^ cells among CD8^+^ T_M_ cells and their expression of CCL3 and IFNγ after challenge with *Lm*-gB or *Lm-*LLO_S92_ are shown. (**E**) Mice grafted with OT-I cells were immunized with *VSV*-Ova and ~6 weeks later challenged with *Lm* or *Lm*-Ova. Frequencies of chemokine^+^ and IFNγ^+^ cells among OT-I T_M_ cells after challenge are shown. Panels pool data from either three (A and C) or two (B, D, and E) independent replicate experiments with *n* = 4 to 8 mice with *P* values (*P < 0.05, **P < 0.005, ***P < 0.0005, and ****P < 0.0001). MFI, mean fluorescence intensity.

To validate chemokine-encoding gene up-regulation in cognate Ag–stimulated CD8^+^ T_M_ cells (Fig. 1F), we monitored CCL3, CCL4, and XCL1 chemokine accumulation in Ag (OT-I)– versus inflammation (gBT-I)–triggered T_M_ cells from mice primary immunized with *Lm*-Ova-gB and challenged 6 weeks later with *Lm*-Ova for 8, 16, 32, and 72 hours (Fig. 2). As early as ~4 hours after challenge infection, OT-I, but not gBT-I, T_M_ cells accumulated detectable levels of the three chemokines, peaking between 12 and 16 hours after infection with 30 to 40% chemokine^+^ OT-I T_M_ cells (Fig. 2A). As expected (*11*, *12*, *14*, *15*), both T_M_ cells expressed IFNγ independent of cognate Ag stimulation. Substantial levels of chemokines (CCL3) and IFNγ were measured in short-term culture supernatants of splenocytes (without Golgi Plug/Stop) isolated from 8-hour *Lm*-Ova–challenged versus unchallenged mice, indicative of their active secretion (fig. S1A). By 32 hours, chemokine secretion was terminated, and OT-I T_M_ cells underwent robust clonal expansion (Fig. 2B). To further define which subset of CD8^+^ T_M_ cells (*32*) among central [CX3CR1^low^CD27^hi^ (T_CM_)], peripheral [CX3CR1^int^CD27^hi^ (T_PM_)], or effector [CX3CR1^hi^CD27^low^ (T_EM_)] CD8^+^ T_M_ cells produced chemokines and IFNγ, we flow-sorted these populations and incubated them with their cognate Ag in vitro (Fig. 2C and fig. S1B). While both OT-I T_CM_ and T_PM_ accumulated significantly more chemokines and IFNγ than T_EM_ counterparts, they could nevertheless all produce them. To validate results in endogenous non-TCR transgenic CD8^+^ T_M_ cells and for naturally presented epitopes, we immunized WT B6 mice (H2^b^) that also express the K^d^ molecule (B6-K^d^) with *Lm*-gB, allowing for the tracking of both *Lm*-derived LLO_91–99_/K^d^ and p60_217–225_/K^d^ as well as herpes simplex virus 2 (HSV-2)–derived gB_497–505_/K^b^–specific CD8^+^ T_M_ cells, using the corresponding tetramers (Tet) (Fig. 2D). Six weeks after vaccination, mice were challenged with either *Lm*-gB or *Lm*-LLO_S92_ that lacks the LLO_91–99_ epitope, and we monitored endogenous Tet-specific CD8^+^ T_M_ cell production of chemokines in the presence or absence of their respective cognate Ags. After *Lm*-gB challenge, e.g., when all T_M_ cell cognate Ags were present, gB_498–505_/K^b^, p60_217–225_/K^d^, and LLO_91–99_/K^d^ Tet^+^ CD8^+^ T_M_ cells expressed CCL3. However, when mice were challenged with *Lm*-LLO_S92_, inflammation-only–stimulated LLO_91–99_/K^d^-specific and gB_498–505_/K^b^-specific CD8^+^ T_M_ cells expressed IFNγ but no chemokines, while Ag-triggered p60_217–225_/K^d^-specific CD8^+^ T_M_ cells accumulated both CCL3 and IFNγ. We next extended findings to CD8^+^ T_M_ cells induced with a different vaccination model, by immunizing mice grafted with OT-I cells with Ova-expressing VSV (*VSV-*Ova), challenged them 6 weeks later with either *Lm*-Ova or *Lm*, and quantified chemokine and IFNγ production (Fig. 2E). Likewise, upon immunization with *Lm*, CD8^+^ T_M_ cells induced after *VSV* vaccination also induced a rapid and coordinated burst of Ag-dependent chemokines and Ag-independent IFNγ accumulation, peaking at ~16 hours after challenge infection, with 40 to 60% of chemokine/IFNγ^+^ OT-I T_M_ cells. Thus, together, these data establish that across distinct mouse models of immunization (bacteria and virus) and multiple CD8^+^ T cell epitopes, cognate Ag recognition triggers a rapid and early coordinated burst of chemokine production by CD8^+^ T_M_ cells.

### IFN regulatory factor 4 exerts transcriptional control over chemokine production by CD8^+^ T_M_ cells

**Fig. 3.**
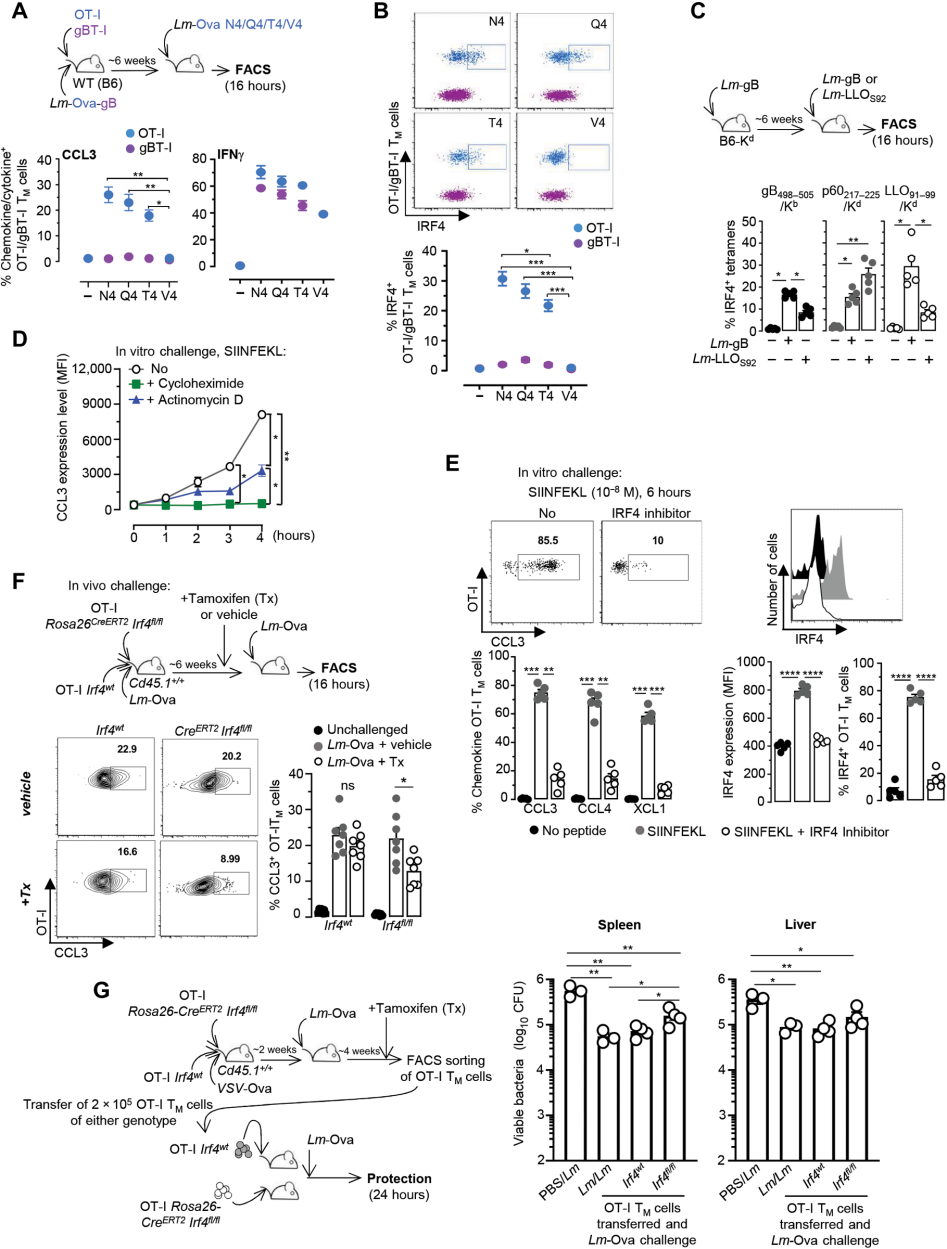
The transcription factor IRF4 orchestrates chemokine production by CD8^+^ T_M_ cells downstream TCR signaling. (**A** and **B**) Mice grafted with OT-I Td^+^ and CD45.1^+/+^ gBT-I cells were immunized with *Lm*-Ova-gB, challenged or not ~6 weeks later for 16 hours with *Lm*-Ova N4, *Lm-*Ova Q4, *Lm-*Ova T4, or *Lm-*Ova V4. Spleen cells were next incubated with GolgiPlug/Stop and stained for cell surface CD8, CD3, CD45.1, and CCL3 and IFNγ (A) or IRF4 (B). Proportions of OT-I or gBT-I T_M_ cells expressing indicated intracellular markers are shown, with representative overlaid dot plots of IRF4 intracellular staining in OT-I and gBT-I T_M_ cells (B). (**C**) B6-K^d^ mice were immunized with *Lm*-gB and challenged ~6 weeks later with *Lm*-gB or *Lm*-LLO_S92_. Polyclonal CD8^+^ T_M_ cells were quantified using indicated Tet. Bar graphs indicate the proportion of IRF4^+^ cells among tetramer^+^ (Tet^+^) cells. (**D** and **E**) Splenocytes from 6-week-immunized mice as depicted in (A) were incubated with SIINFEKL with or without either cycloheximide or actinomycin D (D) or with the IRF4 inhibitor SCG-CBP30 (E) and further stained. Graphs show the proportions and/or expression level of indicated chemokine^+^ and IRF4^+^ OT-I T_M_ cells. (**F**) *Rosa26^CreERT2^Irf4^flox/flox^Cd45.2^+/+^* and *Irf4^wt^Cd45.1^+/−^* OT-I cells were cotransferred to *Cd45.1^+/+^* WT mice and immunized with *Lm*-Ova. Six weeks later, mice received Tx or vehicle before *Lm*-Ova challenge infection. CCL3 expression was determined 16 hours later. (**G**) *Rosa26^CreERT2^Irf4^flox/flox^ Cd45.2^+/+^* and *Irf4^wt^Cd45.1^+/−^* OT-I cells were cotransferred to *Cd45.1^+/+^* WT mice, immunized with *VSV*-Ova, and boosted with *Lm*-Ova 2 weeks later. After 4 weeks, mice received Tx or vehicle before sorting spleen OT-I T_M_ cells. A total of 2 × 10^5^ OT-I T_M_ cells of either genotype were transferred to recipient mice, challenged with *Lm*-Ova, and spleens/livers were plated to enumerate *Lm* titers. Control groups included age-matched WT mice either primary [phosphate-buffered saline (PBS)/*Lm*)] or secondary (*Lm/Lm*) challenged with *Lm*. Panels pool the result of two independent replicate experiments with *n* = 6 (A and B), 5 (C to E), and 7 (F) mice. In (G), experiment was performed once with *n* = 3 to 4 mice per group (one symbol, one mouse). *P* values are indicated. ns, not significant. *P < 0.1, **P < 0.01, ***P < 0.001, and ****P < 0.0001.

Cognate Ag stimulation induces up-regulation of CCL3, CCL4, and XCL1 chemokine-encoding genes in CD8^+^ T_M_ cells and their subsequent secretion, suggesting a common transcriptional mechanism of regulation. Our transcriptomic analysis revealed several genes involved in the regulation of transcription, such as the transcription factor IRF4 (IFN regulatory factor 4), that are up-regulated upon cognate Ag recognition. Since IRF4 expression in T cells is directly proportional to the strength of TCR signals (*33*, *34*), we expected that, if IRF4 controlled chemokine expression, lowering TCR signaling should lead to a proportional and concomitant loss of IRF4 and chemokine expression by CD8^+^ T_M_ cells. To test this possibility, we used *Lm* expressing three different Ova_257–264_ (SIINFEKL) altered peptide ligands (APLs) in which the original asparagine amino acid in position 4 of the peptide (N4) is replaced by either a glutamine (Q4), a threonine (T4), or a valine (V4), decreasing OT-I TCR signaling by factors of ~20, 70, and 700 times, respectively (*35*). Mice grafted with OT-I and gBT-I cells were immunized with *Lm*-Ova-gB and, 6 weeks later, either left unchallenged or challenged with *Lm* expressing each Ova APL or control *Lm*-Ova (N4). We next monitored the secretion of chemokines and IFNγ 16 hours later (Fig. 3A and fig. S1C). Decreasing OT-I TCR signaling led to a proportional loss of chemokine-producing T cells (CCL3, CCL4, and XCL1), which also directly correlated with the loss of IRF4 expression (Fig. 3B). As expected, inflammation-stimulated gBT-I T_M_ cells neither produced chemokines nor up-regulated IRF4, while IFNγ production remained comparable across all challenge conditions, in both cognate Ag (OT-I)– and inflammation (gBT-I)–triggered CD8^+^ T_M_ cells. To ensure that IRF4 up-regulation during endogenous pathogen-specific polyclonal CD8^+^ T_M_ cell response was comparable to that of OT-I TCR transgenic T cells, we next used the same immunization/challenge approach as in Fig. 2C. Here, gB_498–505_/K^b^, p60_217–225_/K^d^, and LLO_91–99_/K^d^ Tet^+^ CD8^+^ T_M_ cells underwent the most robust up-regulation of IRF4 expression during challenge infection in the presence of their respective cognate Ag (Fig. 3C). These results collectively indicate a direct correlation between the strength of TCR signaling and the proportion of chemokine-producing CD8^+^ T_M_ cells. Furthermore, “in vitro challenge” of OT-I T_M_ cells isolated from *Lm-*Ova–immunized mice with the SIINFKEL epitope in the presence or absence of broad inhibitors of either translation (cycloheximide) or transcription (actinomycin D) suggested that most of the CCL3 in Ag-stimulated T_M_ cells was being rapidly transcribed (>60%) (Fig. 3D), while only a smaller proportion was stored as mRNA (~30%) but none as protein, a result also consistent with recent reports (*36*, *37*). Together, these data support the hypothesis that IRF4 acts as a transcriptional regulator of chemokine expression downstream of TCR signaling.

To establish whether IRF4 controls CCL3, CCL4, and XCL1 chemokine expression in CD8^+^ T_M_ cells, we blocked IRF4 in OT-I T_M_ cells and quantified their production of chemokines. We first challenged OT-I T_M_ cells isolated from *Lm-*Ova–immunized mice with the SIINFKEL peptide in vitro challenge in the presence of the chemical inhibitor SCG-CBP30, which selectively inhibits bromodomain-containing transcription factors such as IRF4 (Fig. 3E). We found that IRF4 expression in OT-I T_M_ cells was prevented and the proportion of chemokine^+^ cells was significantly decreased (by ~80%) compared to incubation with peptide only. To next confirm and validate findings in vivo, we generated OT-I^+^ *Rosa26^CreERT2^Irf4^flox/flox^* mice in which *Irf4* could be inducibly deleted in OT-I T_M_ cells (Fig. 3F). Naïve *Rosa26^CreERT2^Irf4^flox/flox^* or WT *Cd45.1/2* OT-I cells were cotransferred to WT *Cd45.1^+/+^* recipient mice and then immunized with *Lm*-Ova, and 6 weeks later, mice received either tamoxifen (Tx) or vehicle for 5 days before *Lm*-Ova recall infection. In Tx-treated groups, the proportion of *Rosa26^CreERT2^Irf4^flox/flox^* OT-I T_M_ cells secreting chemokines (CCL3) was significantly decreased compared to that of WT counterparts (by ~45%), yet both of these genotypes secreted comparable amounts of CCL3 in mock-treated mice. Expression levels of IRF4 were also diminished in Tx-treated but not mock-treated *Rosa26^CreERT2^ Irf4^flox/flox^* OT-I T_M_ cells, further validating this result (fig. S1D). Last, we determined the contribution of CD8^+^ T_M_ cell–derived chemokines to immunized host protection. For this, we tested whether deleting IRF4 in OT-I T_M_ cells, which prevents rapid chemokine secretion, altered their ability to confer protection to challenged mice compared to nondeleted counterpart (Fig. 3G). WT recipient mice were cotransferred with *Rosa26^CreERT2^Irf4^fl/fl^* or *Irf4^fl/fl^* OT-I cells that were immunized with *VSV*-Ova the next day. After 2 weeks, mice were boosted with *Lm*-Ova and, 4 weeks later, treated with Tx daily for 5 days. Then, both genotypes of OT-I T_M_ cells were flow-sorted and transferred to new recipient mice further challenged with *Lm*-Ova. Bacterial titers were enumerated 24 hours later and showed that memory cells lacking IRF4 conferred ~50% (in spleen and liver) less protection than that of WT counterparts. Thus, cognate Ag signaling via IRF4 is required to achieve full protection. In conclusion, our data establish that IRF4 contributes to the transcriptional regulation of the coordinated and simultaneous burst of CCL3, CCL4, and XCL1 chemokines produced by Ag-activated CD8^+^ T_M_ cells in vitro and in vivo and subsequent immunized host protection.

### Monocyte clustering occurs independently from cognate Ag or IFNγ signaling

**Fig. 4.**
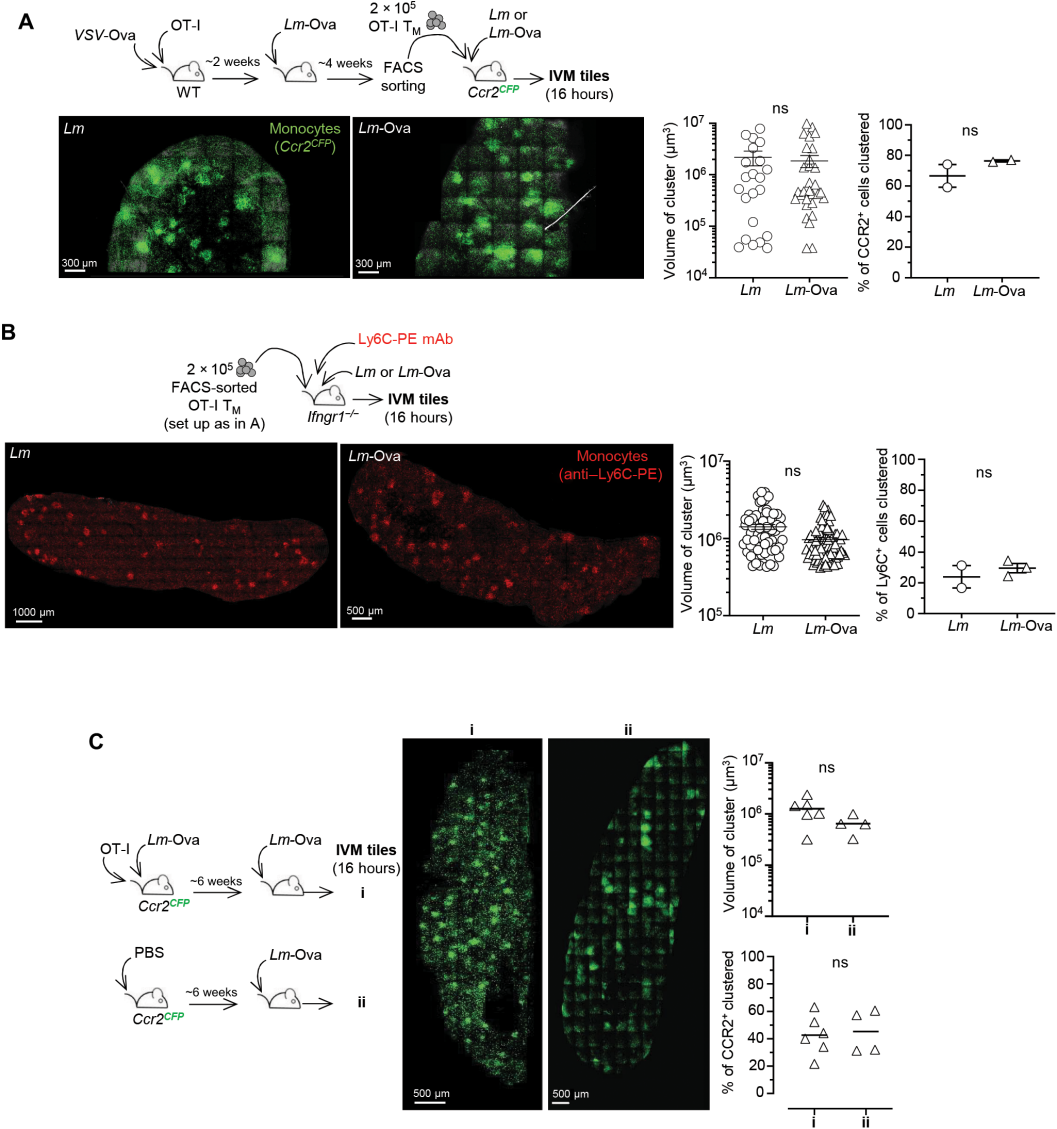
CCR2^+^Ly6C^+^ monocyte clusters form in the splenic RP independent from cognate Ag, IFNγ, and CD8^+^ T_M_ cells. (**A** and **B**) A total of 2 × 10^5^ OT-I T_M_ flow-sorted cells generated upon immunization with 2 × 10^5^ plaque-forming units (PFUs) of *VSV*-Ova and challenge with 10^6^ *Lm*-Ova were transferred to naïve *Ccr2^CFP^* (A) or *Ifngr1^−/−^* (B) recipient mice subsequently challenged with 10^6^ *Lm* or *Lm*-Ova for 16 hours. Representative intravital imaging microscopy (IVM) tiles of reconstructed mouse spleens with CCR2^+^ (A) or Ly6C^+^ (B) monocytes in spleen’s RP. In (B), *Ifngr1^−/−^* mice were also coinjected with the anti–Ly6C-PE Ab (10 μg). Graphs in (A) and (B) show the volume of individual clusters and the average proportion of clustered CCR2^+^Ly6C^+^ monocytes in each mouse spleen analyzed across two independent replicate experiments (*n* = 2 to 3). (**C**) Mice were transferred with OT-I cells and primary and secondary challenged with *Lm*-Ova (i) or only primary immunized with *Lm*-Ova. Ccr2^CFP^ monocytes are in green, and scales are indicated. Representatives IVM tiles of reconstructed *Ccr2^CFP^* mouse spleens. Graphs show the average volume of clusters and proportion of clustered monocytes in each mouse spleen analyzed.

We previously showed that CD8^+^ T_M_ cell–mediated control of *Lm* growth during recall infection occurs within only a few hours (~6 to 8 hours) and correlates with their rapid localization with clustered CCR2^+^Ly6C^+^ monocytes and neutrophils in the splenic red pulp (RP) of infected mice, at portal of bacterial entry (*20*, *27*, *38*). Thus, we hypothesized that CD8^+^ T_M_ cell–derived chemokines produced in response to cognate Ag recognition orchestrate monocyte homing and clustering to rapidly prevent pathogen spreading and help deliver local IFNγ. To gain deeper understanding of this process, we first monitored the kinetics of Ly6C^+^ monocyte clustering in the RP of *Lm*-vaccinated mice undergoing a recall infection (fig. S2A). *Ccr2*^CFP^ mice, in which all CCR2^+^Ly6C^+^ monocytes express the cyan fluorescent protein (CFP) reporter protein, were grafted with OT-I cells and immunized with *Lm-*Ova. Six weeks later, mice were left unchallenged or challenged with *Lm*-Ova, and spleens were harvested 3, 6, 16, and 40 hours later for whole-organ tile reconstruction using multiphoton laser scanning microscopy that only enables to visualize splenic RP (fig. S2A). Already by 3 hours after challenge infection, few clusters of CCR2^+^Ly6C^+^ monocytes were detected, with proportions increasing from 6 hours to peaking at 16 hours and with some clusters still present by 40 hours. Notably, peak clustering of monocytes at 16 hours correlated with that of chemokines produced by the T_M_ cells (Fig. 2A and fig. S2A). To better investigate the role of CD8^+^ T_M_ cells and cognate Ag in CCR2^+^Ly6C^+^ monocyte cluster formation, we next adoptively transferred OT-I T_M_ cells in *Ccr2*^CFP^ WT mice subsequently challenged with *Lm* or *Lm*-Ova and monitored monocyte clustering in spleen RP (Fig. 4A). For this, we took advantage of a heterologous prime/boost immunization strategy of mice grafted with OT-I cells, primed with *VSV*-Ova, and challenged with *Lm*-Ova to generate sufficiently high numbers of OT-I T_M_ cells for purification and transfer. Whether cognate Ag was present, the proportion and volume of monocyte clusters at the peak (16 hours) remained comparable, a result that we also confirmed in WT *Ccr2*^CFP^ mice grafted with OT-I cells, immunized with *VSV*-Ova, and challenged with either *Lm* or *Lm*-Ova 6 weeks later (fig. S3A). Next, since IFNγ signaling is an essential contributor to vaccinated host protective responses (*20*), we tested whether it may direct CCR2^+^Ly6C^+^ monocyte clustering. For this, we adoptively transferred OT-I T_M_ cell in *Ifngr1^−/−^* mice that we next challenged with *Lm* or *Lm*-Ova (Fig. 4B). Since CCR2^+^Ly6C^+^ monocytes in *Ifngr1^−/−^* mice did not express CFP, we tracked them using intravenous injection of anti–Ly6C–PE (phycoerythrin) monoclonal antibody (mAb) 16 hours before imaging, which colabels all detectable clustered CFP^+^ monocytes (fig. S3B). As before, whether cognate Ag (Ova) and IFNγ signaling were present, the proportion and volume of CCR2^+^Ly6C^+^ monocyte clusters at 16 hours were also comparable. Last, we tested whether CD8^+^ T_M_ cells were required for CCR2^+^Ly6C^+^ monocyte clustering to occur (Fig. 4C). Unimmunized mice challenged with *Lm*-Ova, which do not control the infection compared to immunized counterpart (fig. S2B), developed comparable numbers and volume of CCR2^+^Ly6C^+^ monocyte clusters 16 hours after infection. This result indicated that the presence of immunization-induced memory cells is not essential for monocyte homing and clustering to occur, although they may still alter CCR2^+^Ly6C^+^ monocyte functions. Hence, together, these data establish that CCR2^+^Ly6C^+^ monocyte homing and clustering mostly occur independently of the presence of Ag-specific CD8^+^ T_M_ cells and IFNγ signaling.

### Cognate Ag on DCs, but not monocytes, controls CD8^+^ T_M_ cell production of chemokines and arrest in CCR2^+^Ly6C^+^ monocyte clusters

**Fig. 5.**
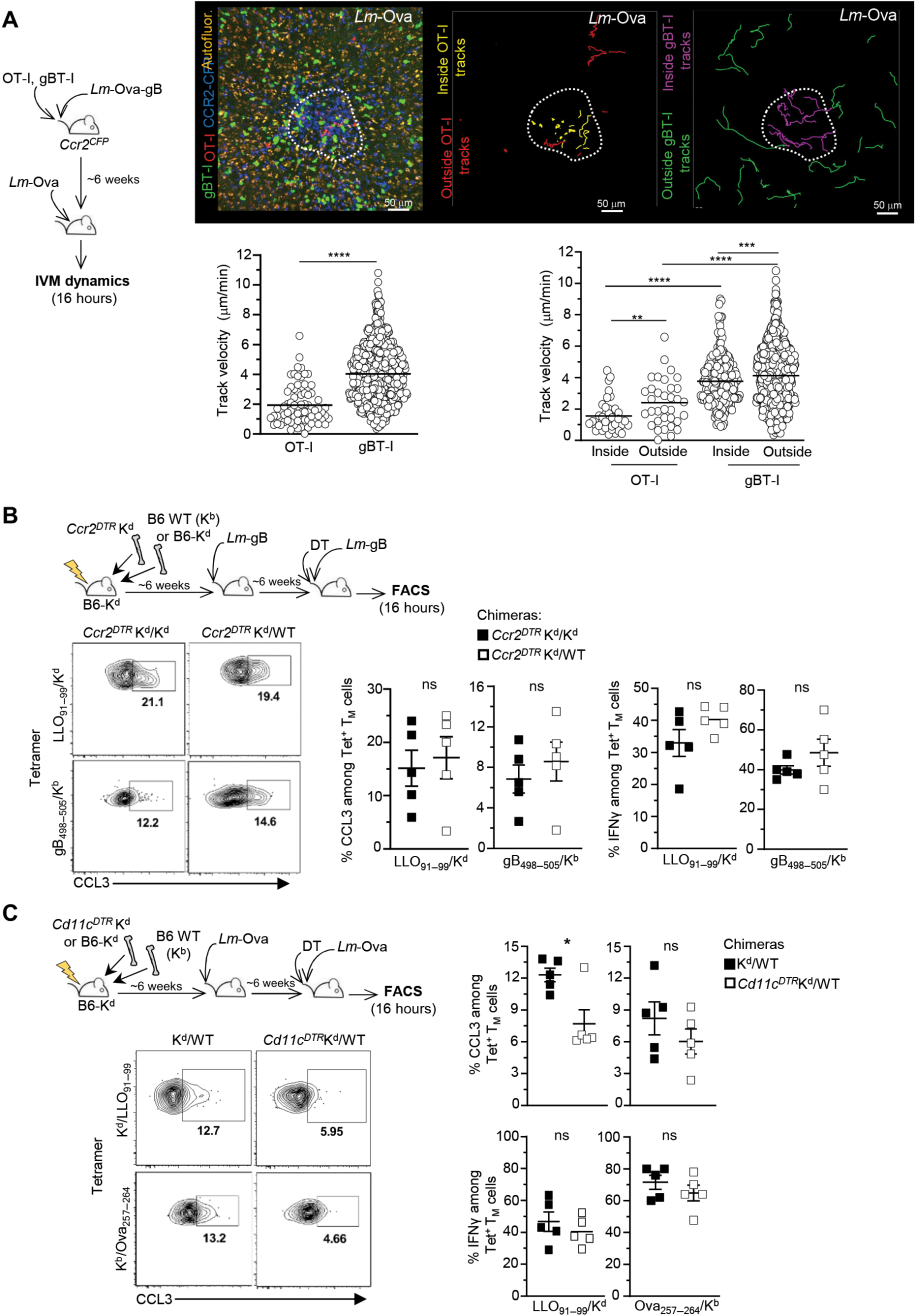
Memory CD8^+^ T cells arrest upon cognate Ag recognition presented by DCs but not CCR2^+^Ly6C^+^ monocytes. (**A**) *Ccr2^CFP^* mice cotransferred with naïve OT-I Td^+^ and gBT-I GFP^+^ cells and immunized with 10^4^ *Lm-*Ova-gB were challenged ~6 weeks later with 10^6^ *Lm*-Ova, and spleens from live mice were exposed and imaged 16 hours later using IVM imaging. A representative image (left) of OT-I (red) and gBT-I (green) T_M_ cell localization in a cluster (white dotted line) of CCR2^+^Ly6C^+^ monocytes (blue) is shown. Autofluorescence appears in yellow. Also shown (center and right images) are OT-I T_M_ cell tracks (outside, red and inside, yellow) and gBT-I T_M_ cell tracks (outside, green and inside, purple) inside/outside the same cluster of CCR2^CFP^ monocytes. Graphs represent the speed of individual OT-I and gBT-I T_M_ cells in the monocyte cluster area (left) and inside/outside the cluster. (**B** and **C**) Lethally irradiated (1200 rads) B6-K^d^ recipient mice were reconstituted with (B) B6-K^d^ or WT B6 (K^b^) and *Ccr2^DTR^* K^d^ BM or (C) *Cd11c^DTR^*K^d^ or K^d^ and WT B6 BM. Six weeks after reconstitution, mice were immunized with 10^4^ *Lm-*gB (B) or *Lm*-Ova (C) and, 6 weeks later, challenged with 10^6^ *Lm-*gB 12 hours after DT treatment. Endogenous CD8^+^ T_M_ cells were monitored using LLO_91–99_/K^d^, gB_498–505_/K^b^, or Ova_257–264_/K^b^ Tet. Graphs show the expression of CCL3 and IFNγ among Tet^+^ cells after challenge. Each symbol corresponds to one individual mouse in one of two replicate experiments, and *P* values are shown. *P < 0.1, **P < 0.01, ***P < 0.001, and ****P < 0.0001.

While CCR2^+^Ly6C^+^ monocyte homing and clustering still occurred in unimmunized mice, these clusters could nevertheless be necessary for a protective recall response in immunized mice. Thus, we pursued the hypothesis that CCR2^+^Ly6C^+^ monocyte clustering is functionally important and that clusters may act as local “hubs” in which CD8^+^ T_M_ cells arrest and deliver IFNγ and other effector molecules to them and to other innate immune cells recruited to these clusters, i.e., neutrophils and natural killer (NK) cells (*20*, *27*). We used intravital imaging microscopy (IVM) of spleen RP in *Ccr2*^CFP^ living mice undergoing a recall infection (Fig. 5A and movies S1 to S3). Mice transferred with OT-I (Td^+^) and gBT-I [green fluorescent protein (GFP)^+^] cells were immunized with *Lm-*Ova-gB and challenged 6 weeks later with either *Lm*-Ova, *Lm*, or *Lm*-Ova-gB. In *Lm*-Ova–challenged mice, in which only OT-I T_M_ cells recognize their cognate Ag, most OT-I T_M_ cells localized in the cluster of monocytes (CFP^+^) and arrested or only exhibited very limited motility (track velocity, 1.93 μm/min) (Fig. 5A, movie S1, and fig. S4A). In contrast, gBT-I T_M_ cells were more motile (track velocity, 4.01 μm/min), but they were enriched in the monocyte clusters similarly to OT-I cell counterparts (fig. S4B). Both T_M_ cells’ speed also decreased inside compared to outside monocyte clusters, collectively suggesting that non–cognate Ag signals affect their homing to and motility in the clusters (Fig. 5A). As expected, in *Lm*-Ova-gB–challenged mice, where both T_M_ cells recognize their cognate Ag, OT-I and gBT-I T_M_ cells arrested in the clusters while simultaneously exhibiting higher motility outside of clusters (movie S2 and fig. S4B). Moreover, in *Lm*-challenged mice in which no cognate Ag was present, both T_M_ cells exhibited the same pattern of enriched localization inside versus outside the clusters and comparable speeds (movie S3 and fig. S4, A and B). Thus, cognate Ag signals induce Ag-specific T_M_ cell arrest in CCR2^+^Ly6C^+^ monocyte clusters where IFNγ is detected in T_M_ cells (*20*, *27*), indicating that a functional interaction between T_M_ cells and CCR2^+^Ly6C^+^ monocytes may occur in these clusters. In addition, the fact that even non–cognate Ag–specific T_M_ cell speed is reduced inside compared to outside of clusters suggests that the clusters are conductive of a qualitatively distinct, possibly hypoxic, local microenvironment (*39*).

Since T_M_ cells arrest in CCR2^+^Ly6C^+^ monocyte clusters in the presence of cognate Ag and T cells arrest in response to Ag recognition (*40*–*42*), we postulated that monocytes may present Ag to them. To test this possibility, we generated mixed bone marrow (BM) chimera mice in which selective elimination of K^d^-dependent cognate Ag presentation by CCR2^+^Ly6C^+^ monocytes can be achieved. Here, lethally irradiated B6-K^d^ mice were reconstituted with *Ccr2^DTR^* K^d^ BM and either (i) B6-K^d^ (K^d^) or (ii) B6 (WT) BM (1:1 ratio), producing *Ccr2^DTR^* K^d^/WT mice and *Ccr2^DTR^* K^d^/K^d^ chimeras. In these mice, diphtheria toxin (DT) injection eliminates CCR2^+^ K^d^ monocytes, while DT receptor (DTR)^−^ (K^d^ or WT) CCR2^+^Ly6C^+^ monocytes remain, respectively (fig. S4C). Chimeras were immunized with *Lm*-gB and treated with DT before *Lm*-gB challenge infection, and we monitored both LLO_91–99_/K^d^ and gB_498–505_/K^b^ Tet^+^ CD8^+^ T_M_ cells for Ag-dependent chemokine (CCL3) and Ag-independent IFNγ production (Fig. 5B). The proportion of Ag-stimulated (CCL3^+^) LLO_91–99_/K^d^ Tet^+^ CD8^+^ T_M_ cells was the same whether CCR2^+^Ly6C^+^ monocytes could present the LLO_91–99_/K^d^ Ag (in DT-treated *Ccr2^DTR^* K^d^/K^d^ chimeras) or not (in DT-treated *Ccr2^DTR^* K^d^/WT chimeras). However, the frequency of IFNγ^+^ cells was equivalent, confirming that LLO_91–99_/K^d^ Tet^+^ CD8^+^ T_M_ cells underwent comparable Ag-independent activation in all groups. No differences in the proportion of CCL3^+^ and of IFNγ^+^ gB_498–505_/K^b^ Tet^+^ CD8^+^ T_M_ cells were measured between the various experimental conditions, ruling out a possible impact of DT-induced deletion on T_M_ cell activation. Thus, Ag presentation by splenic CCR2^+^Ly6C^+^ monocytes is not required for Ag-dependent CD8^+^ T_M_ cell activation during recall infection.

DCs quickly uptake *Lm* (*43*, *44*) and contribute to CD8^+^ T_M_ cell reactivation (*7*). Using K^d^/WT and *Cd11c^DTR^* K^d^/WT chimera mice, in which DT injection eliminates CD11c^+^K^d^ DCs while DTR^−^ (WT or K^d^) CD11c^+^ DCs remain (fig. S4D), we tested whether CD11c^+^ DC presented cognate Ag to T_M_ cells after immunization/challenge with *Lm*-Ova (Fig. 5C). A significant decrease (~40%) in CCL3^+^ CD8^+^ T_M_ cells was only measured for LLO_91–99_/K^d^ but not Ova_257–264_/K^b^ Tet^+^ CD8^+^ T_M_ cells, while the proportion of IFNγ^+^ cells remained equivalent between the different groups of chimeras. Thus, together, these data indicate that splenic CD11c^+^ DCs, but not CCR2^+^Ly6C^+^ monocytes, selectively present cognate Ag to CD8^+^ T_M_ cells and that CCR2^+^Ly6C^+^ monocytes cannot substitute for DCs in this task.

### Cognate Ag stimulation of CD8^+^ T_M_ cells potentiates CCR2^+^Ly6C^+^ monocyte effector functions in the clusters

**Fig. 6.**
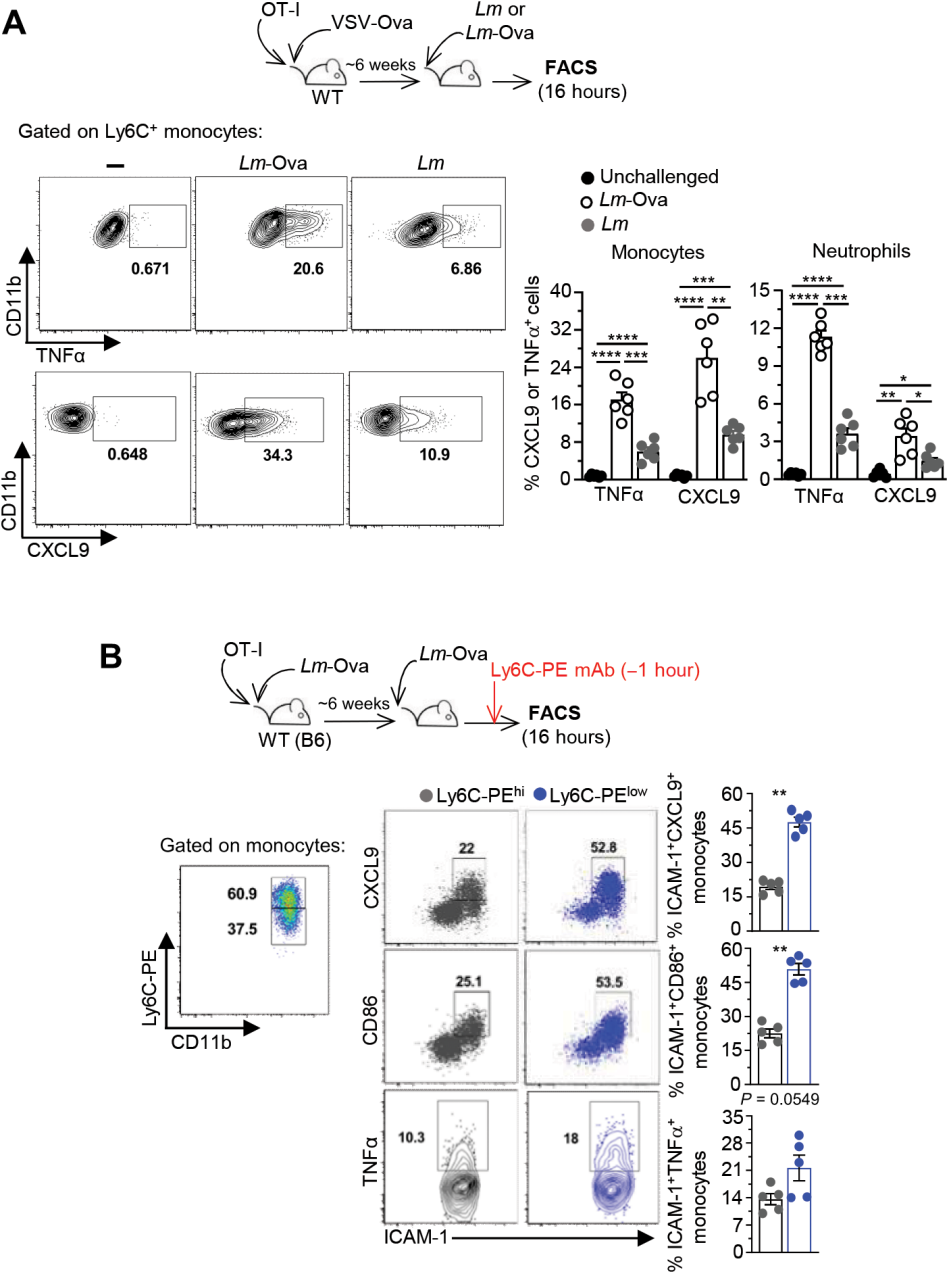
Cognate Ag stimulation enhances CCR2^+^Ly6C^+^ monocyte effector functions in the clusters. (**A**) WT mice transferred with OT-I cells were immunized with 2 × 10^5^ *VSV-*Ova and challenged or not 6 weeks later with 10^6^ *Lm* or *Lm-*Ova. Spleens from 16-hour-challenged or unchallenged mice were harvested, and cells were incubated for 4 to 6 hours with Golgi Plug/Stop before staining for expression of CD11b, Ly6C, and Ly6G cell surface markers and indicated intracellular effector and chemotactic markers. (**B**) *Lm-*Ova (10^4^)–immunized mice were challenged with 10^6^ *Lm*-Ova or not, and 1 hour before euthanasia, 5 μg of Ly6C-PE mAb was intravenously injected to the hosts. Spleens were harvested, and cells were stained for cell surface CD11b, Ly6C-PerCpCy5.5, ICAM-1, CD86, and intracellular TNFα and CXCL9. After gating on Ly6C-PerCpCy5.5^+^ monocytes, Ly6C-PE^hi^ and Ly6C-PE^low^ monocytes were identified and further analyzed for indicated marker expression. Representative FACS dot plots are shown, and bar graphs pool two independent replicate experiments with *n* = 6 (A) and 5 (B) mice. *P* values are indicated. *P < 0.1, **P < 0.01, ***P < 0.001, and ****P < 0.0001.

Cognate Ag enables CD8^+^ T_M_ cell arrest in CCR2^+^Ly6C^+^ monocyte clusters and their concomitant production of a chemokine burst. If, as hypothesized, CD8^+^ T_M_ cell arrest in these clusters is functionally important for local delivery of chemokines and IFNγ, then we predicted that in the presence of cognate Ag, these cells should produce more effector cytokines (Fig. 6A). To test this model, we immunized mice transferred with OT-I T_M_ cells with *VSV*-Ova. Six weeks later, mice were challenged with either *Lm*-Ova or *Lm*, and we monitored TNFα and CXCL9 production in CCR2^+^Ly6C^+^ monocytes and neutrophils. With this experimental setup, the only *Lm*-induced memory cells are the OT-I cells, allowing to specifically assess how the presence of cognate Ag (*Lm*-Ova challenge) affects myeloid cell activation. In the presence of cognate Ag recognition, the proportion of CCR2^+^Ly6C^+^ monocytes and neutrophils producing TNFα and CXCL9 was significantly increased (factor of ~3) compared to mice challenged without cognate Ag (*Lm* challenge), consistent with our proposed model.

To provide further support that CCR2^+^Ly6C^+^ monocyte activation in vivo was spatially restricted to their clusters, we sought to measure the activation of CCR2^+^Ly6C^+^ monocytes inside versus outside the clusters. Similar *Lm*-induced clusters of myeloid cells have been reported to exclude dextrans, suggesting that they were not diffusive (*45*). Therefore, we stained monocytes in vivo using anti–Ly6C-PE mAb injected 1 hour before spleen harvest (1-hour labeling), which, we found, labels all Ly6C^+^ splenocytes that are not within established clusters, in contrast to injecting anti–Ly6C-PE mAb at the time of challenge infection (16-hour labeling), before cluster formation (figs. S5A and S3B). With the 1-hour labeling approach, >90% of CCR2^+^Ly6C^+^ monocytes exhibited equivalent Ly6C-PE staining in unchallenged mice (no clusters), while ~40% of them had lower Ly6C-PE staining in challenged mice, a proportion consistent with that of clustered CCR2^+^Ly6C^+^ monocytes in our microscopy quantifications (Fig. 4A and figs. S5B and S2A). With this approach, we could determine whether CCR2^+^Ly6C^+^ monocyte activation was dependent on localization within clusters during recall infection (Fig. 6B and fig. S5C). A significantly higher proportion of Ly6C-PE^low^ (clustered) compared to Ly6C-PE^hi^ (nonclustered) CCR2^+^Ly6C^+^ monocytes expressed higher levels of intercellular adhesion molecule–1 (ICAM-1), CD86, and intracellular CXCL9 and TNFα, demonstrating that CCR2^+^Ly6C^+^ monocytes undergo robust activation within the clusters, consistent with a spatially restricted delivery of activating cues by arrested CD8^+^ T_M_ cells.

### Blocking Gα_I_-dependent chemotaxis in CX3CR1^+^ cells prevents CCR2^+^Ly6C^+^ spleen monocyte clustering and optimal host protection

**Fig. 7.**
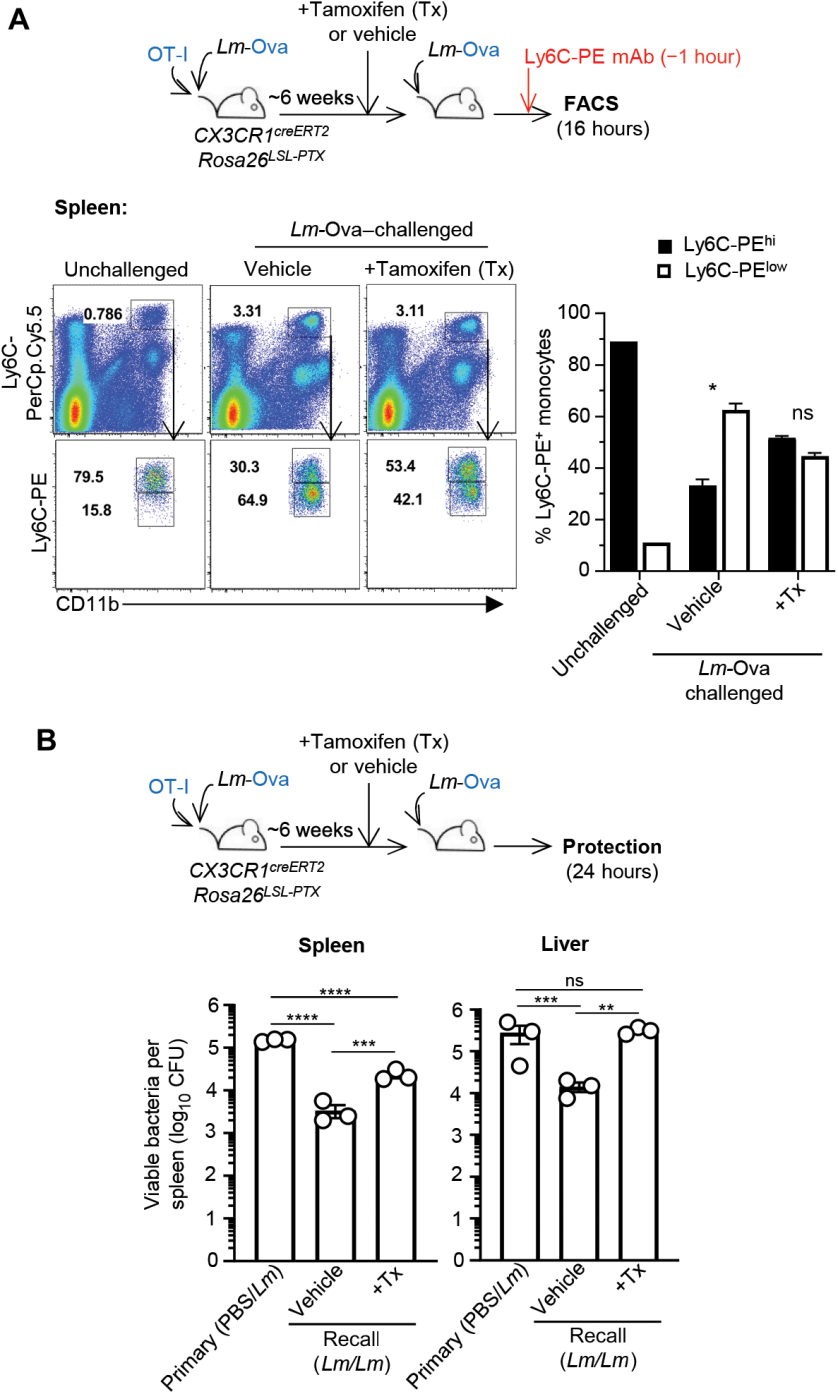
Blocking Gα_I_-dependent chemotaxis in CX3CR1^+^ cells inhibits CCR2^+^Ly6C^+^ splenic monocyte clustering and impairs protection. *CX3CR1^CreERT2^Rosa26^LSL-PTX^* mice transferred with OT-I cells were immunized with 10^4^ *Lm*-Ova. Six weeks later, mice received Tx or vehicle intraperitoneally daily for 5 days before 10^6^ *Lm*-Ova recall infection. (**A**) One hour before harvesting spleens and 16 hours after challenge, 5 μg of Ly6C-PE mAb was injected intravenously to the hosts. Cells were then stained for cell surface CD11b and Ly6C-PerCpCy5.5. After gating on Ly6C-PerCpCy5.5^+^ monocytes, the proportion of Ly6C-PE^hi^ and Ly6C-PE^low^ monocytes was quantified. Representative FACS dot plots are shown, and bar graphs pool two independent replicate experiments with *n* = 6. (**B**) Twenty-four hours after *Lm* infection (10^6^), spleens and livers were harvested and plated to enumerate *Lm* titers. A nonimmunized group of age-matched mice (“primary”) were also infected as control. Bar graphs show one of two independent experiments with *n* = 3 mice. *P* values are indicated. *P < 0.1, **P < 0.01, ***P < 0.001, and ****P < 0.0001.

To further explore whether CCR2^+^Ly6C^+^ monocyte clustering is important for immunized host protection, we took advantage of a mouse model (*46*) in which expression of the pertussis toxin (PTX), which blocks Gα_I_ protein–coupled receptor signal transduction and related chemotaxis, can be induced upon Tx injection in monocytes (Fig. 7A). We used *CX3CR1^ERT2Cre^* mice in which ~50% of CCR2^+^Ly6C^+^ monocytes express the Tx-inducible estrogen receptor T2 (ERT2)-Cre recombinase, crossed to mice that carry a Cre-inducible PTX-encoding gene in the Rosa26 locus (*Rosa26^LoxP-STOP-LoxP(LSL)-PTX^*). Mice were next transferred with OT-I cells, immunized with *Lm-*Ova, and treated with Tx for 5 days before *Lm*-Ova secondary challenge. One hour before euthanizing mice, we injected the anti–Ly6C-PE Ab to label clustered CCR2^+^Ly6C^+^ monocytes in the challenged or unchallenged control mice. As expected, CCR2^+^Ly6C^+^ monocytes were not clustered in unchallenged mice (>80% Ly6C-PE^hi^), while they clustered in vehicle-treated challenged groups (~60% Ly6C-PE^low^). In Tx-treated mice, in which PTX is induced in CX3CR1^+^ cells, clustering was reduced by ~70% compared to vehicle-treated mice (~44% Ly6C^low^), showing that CCR2^+^Ly6C^+^ monocytes required Gα_I_-dependent chemotaxis to cluster. While most of the spleen CCR2^+^Ly6C^+^ monocytes failed to cluster, twice as many CCR2^+^Ly6C^+^ monocytes also could not egress from the BM (*47*) in Tx-treated compared to vehicle-treated mice (fig. S6). We next used this experimental system to evaluate the impact on host protection (Fig. 7B). *CX3CR1^ERT2Cre^Rosa26^LSL)-PTX^* mice transferred with OT-I and either immunized with *Lm*-Ova or injected with phosphate-buffered saline (PBS) were treated with Tx or vehicle 6 weeks later before challenge with *Lm*-Ova. After 24 hours, we harvested and plated spleens and livers and enumerated *Lm* titers. Protected mice exhibited ~50 (spleen) and ~20 (liver) times less bacteria compared to primary challenged mice. Tx-treated mice had ~7 (spleen) and ~19 (liver) times higher bacterial loads than vehicle-treated protected mice. These results show that blocking Gα_I_-dependent chemotaxis in CX3CR1^+^ cells leads to a significant loss of protection in immunized mice undergoing a recall infection, suggesting that splenic CCR2^+^Ly6C^+^ monocyte clustering contributes to protection.

### Cognate Ag stimulation and IFNγ signaling are both required for CD8^+^ T_M_ cell–dependent protection of immunized mice

**Fig. 8.**
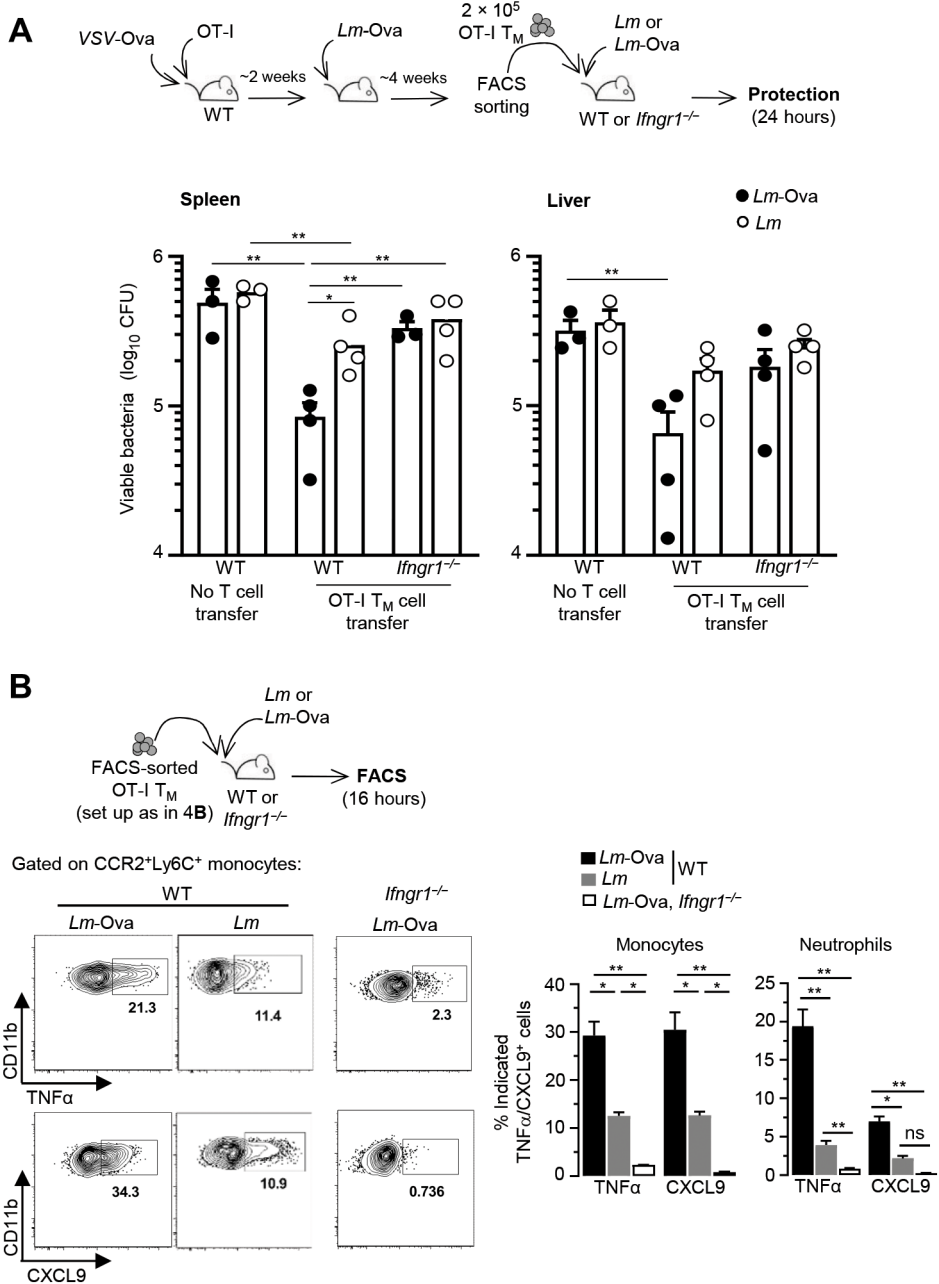
Memory CD8^+^ T cell–mediated protection of vaccinated hosts requires both cognate Ag and IFNγ signaling. A total of 2 × 10^5^ OT-I T_M_ cells induced using the depicted experimental setup (as in Fig. 4B) were transferred in age- and sex-matched WT B6 or *Ifngr1^−/−^* mice, and mice were next challenged with 10^6^ *Lm* or *Lm-*Ova. (**A**) Control groups did not receive OT-I T_M_ cells. Spleens and livers from challenged mice were harvested 24 hours later, and *Lm* colony-forming units (CFUs) were determined after plating. Bar graphs show one of two representative experiments with each symbol corresponding to one individual mouse. (**B**) Spleens from WT or *Ifngr1^−/−^* mice transferred with OT-I T_M_ cells and challenged with 10^6^ of indicated *Lm* were harvested, and cells were stained for expression of cell surface CD11b, Ly6C, and Ly6G and intracellular TNFα and CXCL9. Representative FACS dot plots are shown, and bar graphs pool two representative experiments (*n* = 7 mice) with *P* values indicated. *P < 0.1 and **P < 0.01.

Since both cognate Ag stimulation and IFNγ signaling are required for CD8^+^ T_M_ cell–dependent protection of immunized hosts against challenge infection, we next assessed the relative contribution of both mechanisms. We adoptively transferred OT-I T_M_ cells to naïve WT or *Ifngr1^−/−^* mice that were further challenged with a lethal dose of *Lm* (no cognate Ag) or *Lm*-Ova (with cognate Ag) (Fig. 8A). Control groups did not receive any OT-I T_M_ cells. Bacterial titers in spleens and livers were quantified 24 hours later. While as expected, transfer of OT-I T_M_ cells conferred significant levels of protection to WT recipient mice against *Lm*-Ova challenge (considered 100%), and protection was reduced to ~40% in both organs when challenged with *Lm* (no cognate Ag). We also recapitulated these findings in WT mice primary immunized with *VSV*-Ova and challenged 6 weeks later with either *Lm* or *Lm*-Ova (fig. S7A). However, OT-I T_M_ cell transfer in *Ifngr1^−/−^* mice only conferred modest protection against challenge with *Lm* or *Lm*-Ova, with more than 60% protection loss compared to WT mice. However, in *Lm-*Ova (but not *Lm*)–challenged *Ifngr1^−/−^* mice, OT-I T_M_ cells still efficiently recognized their cognate Ag and produced chemokines (fig. S7B). Consistent with these results, CCR2^+^Ly6C^+^ monocyte and neutrophil production of TNFα and CXCL9 effector cytokine/chemokine in WT or *Ifngr1^−/−^* mice that received OT-I T_M_ cells was significantly reduced when cognate Ag was absent (*Lm* challenge) or IFNγ signaling (*Ifngr1^−/−^*) was disrupted (Fig. 8B). Together, these results indicate that cognate Ag stimulation and IFNγ signaling are both required to achieve optimal protection and that neither of these signals is individually sufficient.

### Memory CD8^+^ T cell–derived chemokines are necessary but not sufficient to activate monocyte effector functions

**Fig. 9.**
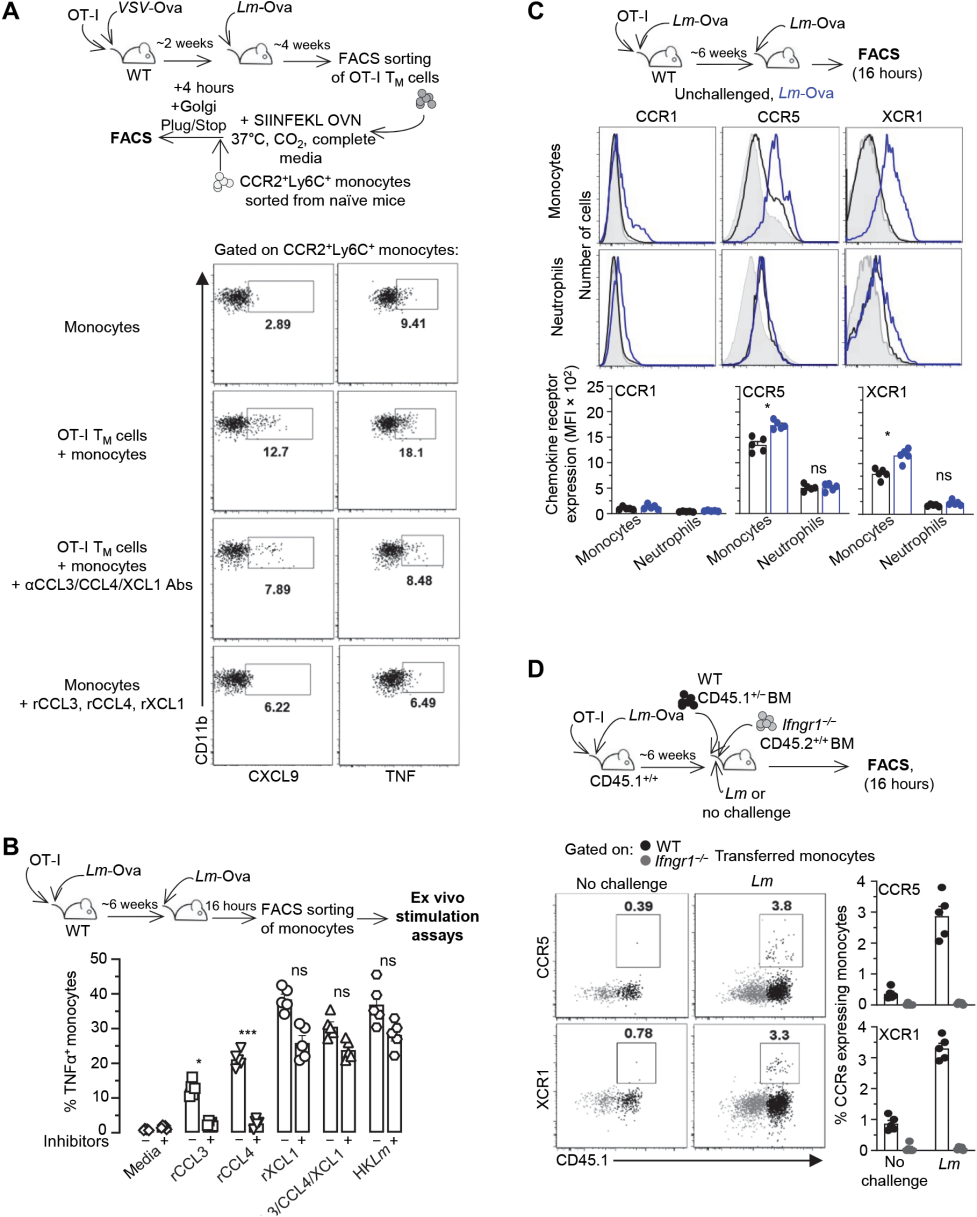
Chemokines secreted by CD8^+^ T_M_ cells enable CCR2^+^Ly6C^+^ monocyte activation in an IFNγ-dependent manner. (**A**) WT mice transferred with OT-I cells were immunized with *VSV*-Ova and boosted with *Lm*-Ova 2 weeks later. After 4 weeks, FACS-sorted OT-I T_M_ cells were stimulated with SIINFEKL peptide (10^−6^ M) OVN in complete media (37°C, CO_2_). The next day, CCR2^+^Ly6C^+^ monocytes, FACS-sorted from a naïve spleen, were added with GolgiPlug/Stop to the T cells with or without CCL3, CCL4, and XCL1 chemokine-neutralizing antisera. Controls included monocytes only with or without the recombinant chemokines. Intracellular accumulation of CXCL9 and TNFα in the monocytes was quantified by FACS 4 hours later. (**B**) CCR2^+^Ly6C^+^ monocytes were FACS-sorted from the spleen of OT-I–transferred immunized mice challenged 6 weeks later with *Lm*-Ova. Recombinant chemokines and HK*Lm* were added or not to purified monocytes with GolgiPlug/Stop. Four hours later, TNFα accumulation was quantified by FACS. (**C**) WT mice transferred with OT-I cells were immunized with *Lm-*Ova and challenged or not 6 weeks later with *Lm-*Ova. Spleens from challenged or unchallenged mice were incubated for 4 to 6 hours with GolgiPlug/Stop before staining for expression of CD11b, Ly6C, and Ly6G cell surface markers and expression of CCR1, CCR5, and XCR1 chemotactic receptors. (**D**) BM from WT (CD45.1^+/−^) and *Ifngr1^−/−^* (CD45.2^+/+^) mice was cotransferred to CD45.1^+/+^ mice immunized with *Lm*-Ova 6 weeks before and challenged with *Lm*. Sixteen hours later, splenocytes were stained for expression of CCR5 and XCR1 on monocytes. Representative FACS dot plots are shown, and bar graphs pool one in two (A) (*n* = 2 mice) or two (B to D) (*n* = 5 to 6 mice) independent replicate experiments. *P* values are indicated. *P < 0.1 and **P < 0.01.

We established in Fig. 3G that, despite Ag-independent production of IFNγ, cognate Ag signaling via IRF4 is required to achieve full protection of immunized hosts. A possible interpretation of this result is that the failure of IRF4-deficient OT-I T_M_ cells to protect is not due to their inability to secrete chemokines but rather to stop in CCR2^+^Ly6C^+^ monocyte clusters to deliver activating IFNγ. Thus, to directly test the role of chemokines in CCR2^+^Ly6C^+^ monocyte activation, we developed an in vitro assay where purified OT-I T_M_ cells and CCR2^+^Ly6C^+^ monocytes were incubated together, with or without chemokine-neutralizing polyclonal antisera (Fig. 9A). Here, OT-I T_M_ cells flow-sorted from primed/boosted mice were incubated overnight (OVN) with their cognate peptide, and the next day, CCR2^+^Ly6C^+^ monocytes, also flow-sorted from naïve WT mice, were added to the culture in the presence of Golgi PLug/Stop, with or without polyclonal neutralizing antisera against each of the three chemokines. A substantially higher proportion of CCR2^+^Ly6C^+^ monocytes produced CXCL9 and TNFα when coincubated with cognate Ag–activated OT-I T_M_ cells than without (factor of 4 and 2, respectively). CCR2^+^Ly6C^+^ monocyte activation was abrogated when cells were incubated in the presence of neutralizing antisera against CCL3, CCL4, and XCL1, demonstrating that memory T cell–derived chemokines are required to trigger monocyte production of TNFα and CXCL9. We next asked whether addition of the CCL3, CCL4, and XCL1 recombinant chemokines, which respectively bind CCR5/CCR1 and XCR1, to CCR2^+^Ly6C^+^ monocytes is sufficient to drive their activation, but they failed to do so (Fig. 9A). However, when CCR2^+^Ly6C^+^ monocytes were isolated from *Lm*-challenged rather than naïve mice, addition of exogenous chemokines enhanced their production of TNFα ex vivo (Fig. 9B and fig. S8, A and B). After incubation with rCCL3, rCCL4, and rXCL1, CCR2^+^Ly6C^+^ monocytes from challenged mice accumulated intracellular TNFα in 15, 20, and 40% of total CCR2^+^Ly6C^+^ monocytes, respectively, and in a dose-dependent manner. Blocking CCR5 and CCR1 with chemical inhibitors during coincubation with the corresponding recombinant chemokines prevented TNFα production by CCR2^+^Ly6C^+^ monocytes, ruling out any CCR1/CCR5-independent activation mechanisms. Incubation with heat-killed *Listeria monocytogenes* (HK*Lm*) induced 40 to 50% of them to express TNFα, a proportion similar to that measured in CCR2^+^Ly6C^+^ monocytes incubated with rXCL1 or the combination of chemokines. In contrast to CCR2^+^Ly6C^+^ monocytes, neutrophils largely failed to respond to chemokine restimulation ex vivo, rather implicating a chemokine-independent mechanism for their activation (fig. S8C). In summary, these data establish that CD8^+^ T_M_ cell–derived chemokines are necessary but not sufficient to promote CCR2^+^Ly6C^+^ monocyte production of effector molecules. Other signals derived from Ag-stimulated CD8^+^ T_M_ cells, most likely IFNγ, are needed in conjunction.

### IFNγ signaling controls up-regulation of CCR5 and XCR1 chemokine receptors, enabling CCR2^+^Ly6C^+^ monocyte responsiveness to chemokines and full activation

Our results show that both IFNγ and antigenic signals, specifically chemokines, are required to achieve optimal host protection (Figs. 3G and 8). We also found that CD8^+^ T_M_ cell–derived signals are essential to make CCR2^+^Ly6C^+^ monocytes responsive to chemokine signals and that CCR2^+^Ly6C^+^ monocytes are only responsive to chemokines after infection (Fig. 9, A and B). Upon challenge infection, we noted that CCR5 and XCR1 expression on CCR2^+^Ly6C^+^ monocytes was significantly increased (Fig. 9C). Thus, we further hypothesized that IFNγ signaling triggers chemokine receptor up-regulation on CCR2^+^Ly6C^+^ monocytes, making them responsive to the chemokines released upon cognate Ag stimulation. We tested this idea by cotransferring WT and *Ifngr1^−/−^* BM into WT recipient mice immediately challenged with *Lm* and monitored CCR5 and XCR1 chemokine receptor expression 16 hours later (Fig. 9D). While we could detect CCR5- and XCR1-expressing WT CCR2^+^Ly6C^+^ monocytes, *Ifngr1^−/−^* monocytes failed to up-regulate these receptors, indicating that IFNγ signaling controls cell surface up-regulation of CCR5 and XCR1 on CCR2^+^Ly6C^+^ monocytes, making them responsive to CD8^+^ T_M_ cell–derived chemokines. Hence, these results collectively support a model where IFNγ signaling in CCR2^+^Ly6C^+^ monocytes enables up-regulation of CCR5 and XCR1 chemokine receptors, making them responsive to CCL3, CCL4, and XCL1 chemokines that orchestrate their production of TNFα, a cytokine absolutely required for host protective memory responses against secondary *Lm* infection (*17*, *21*).

## DISCUSSION

**Fig. 10.**
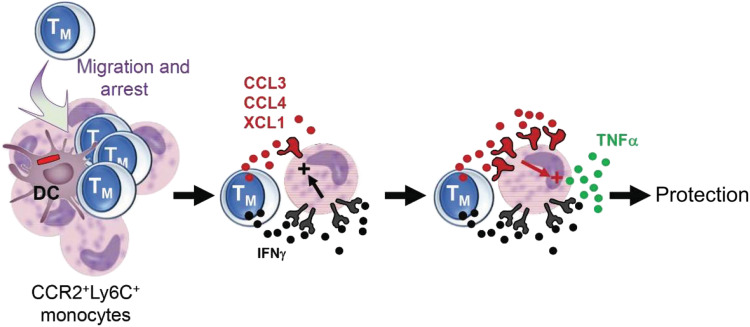
Mechanism of early CD8^+^ T_M_ cell–mediated protection. CD8^+^ T_M_ cells arrest upon cognate Ag recognition presented by DCs in CCR2^+^Ly6C^+^ monocyte splenic RP clusters. Upon local delivery of IFNγ, monocytes up-regulate chemokine receptors (CCR5 and XCR1) and become responsive to cognate Ag–secreted chemokines (CCL3, CCL4, and XCL1). Chemokines then promote CCR2^+^Ly6C^+^ monocyte activation and TNFα production promoting microbicidal effector functions and host protection.

This study provides an in-depth cellular and molecular analysis of how cognate Ag orchestrates and programs the activation of CD8^+^ T_M_ cells for rapid protection against a recall infection in vaccinated hosts in vivo. We show that cognate Ag recognition by CD8^+^ T_M_ cells leads to a broad gene expression program targeting multiple pathways within only a few hours after stimulation. We also highlight that IRF4, downstream and proportional to TCR signaling strength, transcriptionally controls the most significantly up-regulated cluster of genes in cognate Ag–stimulated CD8^+^ T_M_ cells that encode for the chemotactic molecules CCL3, CCL4, and XCL1. Production of these chemokines requires Ag presentation by CD11c^hi^ DCs and cannot be substituted by CCR2^+^Ly6C^+^ monocytes. We reveal that cognate Ag recognition enables CD8^+^ T_M_ cell arrest in splenic RP clusters of CCR2^+^Ly6C^+^ monocytes for the local delivery of IFNγ and chemokines, efficiently restraining microbial pathogen spreading and growth. Our results support a model in which CD8^+^ T_M_ cell–derived IFNγ enables immunized host protection by controlling CCR2^+^Ly6C^+^ monocyte responsiveness to chemokines. Chemokines, in turn, signal to induce TNFα and CXCL9 production by the monocytes. These results suggest a refined model for IFNγ-dependent protection in which CD8^+^ T_M_ (i) IFNγ enables protection by enhancing chemokine signaling and (ii) chemokines act as key effector molecules activating CCR2^+^Ly6C^+^ monocytes, a role that is distinct from their usual role in chemotaxis (Fig. 10).

The current results highlight the importance of rapid microbial pathogen containment, a notion that has been elegantly illustrated in prior reports (*24*, *25*). First-line cellular responders such as splenic marginal zone and LN subcapsular macrophages were reported to rapidly uptake and/or sense microbial pathogens (bacteria and viruses) to subsequently provide chemotactic cues that attract prepositioned memory—but not naïve—CD8^+^ T cells rapidly to the sites of infection. Consistent with these results, intravenously inoculated *Lm* bacteria are rapidly cleared from the blood by marginal zone CD169^+^ macrophages and DCs localized in the splenic RP (*27*, *43*, *44*, *48*, *49*). Following rapid pathogen capture by tissue-resident sentinel cells, a body of evidence suggests that CD8^+^ T_M_ cells home to infectious foci via CXCR3 and/or CCR5 and associated CXCL10, CXCL9, and CCL5 chemokines produced in response to local inflammatory cues such as IFNs (*50*–*52*). A large majority of CD8^+^ T_M_ cells express CXCR3 and CCR5 and thus can be readily mobilized for rapid migration, independent from cognate Ag encounter (*26*). These cells can produce IFNγ in response to cytokines (*11*, *12*, *14*), further increasing local chemokine levels in a feedforward positive loop leading to the rapid amplification of the CD8^+^ T_M_ cell response. We report in the current study that cognate Ag recognition promotes a broad activation program in CD8^+^ T_M_ cells, which includes the early expression of a potent set of chemokines. This finding led us to propose that cognate Ag–triggered CD8^+^ T_M_ cells would amplify the initial chemotactic cues and act as powerful recruiting orchestrators of both adaptive and innate immune cells, setting the stage for more effective microbial pathogen clearance. Unexpectedly, however, our results did not support such a model. Rather, we revealed that CCR2^+^Ly6C^+^ monocytes form clusters in the splenic RP independently from cognate Ag and CD8^+^ T_M_ cells, most likely in response to other infection-driven chemotactic cues. In addition to chemotaxis, adhesion molecules such as ICAM-1, CD11b, and CD44 could be mediating CCR2^+^Ly6C^+^ monocyte trafficking to sites of infection as it was shown in the liver of primary *Lm*-infected mice (*53*). Consistent with this idea, we also noted a strong up-regulation of ICAM-1 on clustered monocytes in spleen RP. Using IVM imaging, we further revealed that, as expected (*40*–*42*), CD8^+^ T_M_ cells arrest upon cognate Ag recognition, which occurs in CCR2^+^Ly6C^+^ monocyte clusters, where they promote their activation through the localized delivery of chemokines and IFNγ. Rather than acting as chemoattractants, the CD8^+^ T_M_ cell–derived chemokines directly enable CCR2^+^Ly6C^+^ monocyte effector functions. While chemokines are necessary, they are not sufficient. IFNγ enables CCR2^+^Ly6C^+^ monocytes responsiveness to the chemokines. These data collectively support a model where CD8^+^ T_M_ cells, through localized delivery of IFNγ and chemokines, function to license and boost monocyte microbicidal function in a targeted manner within monocyte clusters, rather than as initial orchestrators of the early steps of the recall response. Ag presentation also selectively occurs on DC and cannot be substituted by CCR2^+^Ly6C^+^ monocytes.

In a previous study using IVM imaging to explore *Lm* infection foci that form in subcapsular DCs (scDCs) of the splenic RP following primary infection (day 5 after infection), *Lm*-specific effector CD8^+^ T cells were shown to migrate to sites of infection where a mixture of myelomonocytic cells (MMCs), which include CCR2^+^Ly6C^+^ monocytes and neutrophils, accumulates (*39*). These MMCs markedly reduced blood flow access to the sites of infection and restricted *Lm* growth. *Lm*-specific CD8^+^ T cells were also shown by IVM imaging to undergo both Ag-dependent arrest and Ag-independent reduced motility in the scDC/MMC *Lm*-containing clusters, similarly to our observations. However, this study did not address the role that arrested effector CD8^+^ T cells may play in these clusters. Disappearance of *Lm* was associated with effector CD8^+^ T cells regaining motility, but evidence for direct *Lm*-infected killing could not be documented. Together with the large dependence on MMC for *Lm* clearance, these data suggested that, like in the setting of the recall response, noncytolytic T cell–dependent effector mechanisms were essential. In addition to promoting the local expression of microbicial activities in clustered CCR2^+^Ly6C^+^ monocytes, it seems therefore conceivable that the delivery of effector molecules by Ag-arrested CD8^+^ T_M_ cells may also restrict permeability and blood flow in these clusters to ultimately enhance rapid and effective *Lm* containment and killing.

*Lm* killing and vaccinated host protection during recall infection require TNFα, which CCR2^+^Ly6C^+^ monocytes are a major source (*17*, *21*, *54*). TNFα directly triggers microbicidal ROS both from CCR2^+^Ly6C^+^ monocytes and neutrophils, and ROS promotes antimicrobial autophagy (*38*). While we previously showed that IFNγ signaling to CCR2^+^Ly6C^+^ monocytes induces TNFα production by these cells (*20*), we now refine this model by reporting that IFNγ signaling, while necessary, is not sufficient. IFNγ-primed CCR2^+^Ly6C^+^ monocytes become highly responsive to chemokine signals by up-regulating their chemokine receptors, enabling chemokine signals to trigger enhanced microbicidal functions for rapid *Lm* clearance. The fact that both IFNγ and chemokines need to be spatially and temporally targeted on CCR2^+^Ly6C^+^ monocytes at sites of infection to promote their effector responses may represent an evolutionarily conserved mechanism to prevent systemic tissue damages to the host. While we did not monitor neutrophil dynamics here, neutrophils are well known to be massively recruited and undergo activation in infected spleens, and we and others have previously shown that they cluster with CCR2^+^Ly6C^+^ monocytes and CD8^+^ T_M_ cells at infection foci (*16*, *20*, *27*). However, and in contrast to CCR2^+^Ly6C^+^ monocytes, neutrophils neither express nor up-regulate high levels of CCR1, CCR5, or XCR1, suggesting that CD8^+^ T_M_ cell–derived chemokines are unlikely to account for neutrophil activation in this setting. Fine-tuning of CCR2^+^Ly6C^+^ monocyte activation in response to local chemokine levels and chemokine receptor up-regulation may regulate their ability to secrete TNFα, which directly promotes ROS production and pathogen killing.

Another important finding in our study relates to the rapid, transcriptionally controlled, and coordinated production of CCL3, CCL4, and XCL1 chemokines by CD8^+^ T_M_ cells induced upon vaccination with both *Lm* and *VSV* in response to cognate Ag recognition. These results are consistent with two recent reports that used multiple models of acute and chronic infections, as well as ex vivo stimulation assays, outlining that the robust chemokine signature is a key and important feature of both Ag-stimulated effector and CD8^+^ T_M_ cells (*36*, *37*). CD8^+^ T_M_ cells undergoing repetitive in vivo stimulations were also reported to significantly up-regulate genes encoding for these chemokines (*55*). Our study further reveals that CCL3, CCL4, and XCL1 chemokines produced by cognate Ag–stimulated CD8^+^ T_M_ cells are under the control of the IRF4 transcriptional regulator, a known amplifying rheostat downstream of TCR signaling that has been shown to control the size of T cell clonal expansion (*33*). On the basis of our data, IRF4 may also enable the graded production of these chemokines by CD8^+^ T_M_ cells, proportionally to the strength of TCR signaling. Further investigations would be needed to determine whether this involves direct IRF4 transcriptional regulation where IRF4 binds to the promoter or enhancer regions of the chemokine-encoding genes. This is likely to represent a mechanism to limit tissue-associated damages, when weak epitopes are presented to Ag-specific CD8^+^ T_M_ cells. In the context of strong epitope stimulation, however, our results establish that chemokines are secreted concomitantly to CD8^+^ T_M_ cell arrest in CCR2^+^Ly6C^+^ monocyte clusters, promoting their increased production of TNFα and CXCL9 both in vitro and in vivo. These findings also highlight that chemokines can trigger innate immune cell effector functions, delineating a role distinct from usual chemotaxis.

While our study focuses on systemic and secondary lymphoid organ (SLO)-derived CD8^+^ T_M_ cell responses, multiple evidence suggest that the current mechanisms are also relevant in the context of tissue-resident CD8^+^ T_M_ cell responses. In several models of viral infection (skin, vagina, and lung), T_RM_ cells—both CD8^+^ and CD4^+^—quickly initiate and orchestrate a rapid mucosal response upon cognate Ag encounter, through local production of IFNγ and subsequent CXCL9 (*28*–*30*, *51*, *56*, *57*). As discussed earlier, CXCL9 enables migration of more circulating T_M_ cells to sites of infection, enhancing the activation of local DCs and NK cells and the establishment of an IFNγ-driven antiviral state providing broad protective immunity against unrelated microbial pathogens. In these studies, reactivation of CD8^+^ T_M_ cells and the production of activating IFNγ required cognate Ag recognition, yet many reports monitoring systemic CD8^+^ T_M_ cells have also established that CD8^+^ T_M_ cells in SLO undergo cytokine-mediated activation (*11*, *13*–*15*, *26*). This seemingly discrepant result may be a reflection of tissue-specific mechanisms. It was recently shown that LN CD8^+^ T_M_ cells strictly require cognate Ag to be presented by XCR1^+^ DCs, while lung T_RM_ cells can be reactivated by both hematopoietic and nonhematopoietic cells (*8*). Here, cognate Ag presentation by hematopoietic versus nonhematopoietic-derived cells to CD8^+^ T_RM_ cells was also proposed to dictate distinct functional outcomes with hematopoietic-derived Ag-presenting cells restraining an excessive inflammatory program in CD8^+^ T_RM_ cells, presumably as a safeguard mechanism against collateral tissue damages. Nonhematopoietic Ag presentation was associated with a proliferative program and largely prevented cytokine-mediated activation of CD8^+^ T_RM_ cells. Note that this study used the Nur77^GFP^ reporter system, a readout of TCR-dependent cognate Ag stimulation, thereby only focusing on early Ag-dependent CD8^+^ T_M_ cell expression programs. Other reports using complex biological readouts (e.g., proliferation and protection) have supported a more prominent role for recruited or tissue-resident DCs in the reactivation of CD8^+^ T_RM_ cells, raising the possibility that different memory cell–intrinsic mechanisms of regulation may be specifically programmed upon DC-mediated versus nonhematopoietic cell–mediated activation (*9*, *10*).

In conclusion, this work provides a comprehensive analysis of cognate Ag–induced early transcriptional and functional changes in reactivated CD8^+^ T_M_ cells and how these changes enable the rapid control of microbial pathogen in vaccinated hosts in situ. Perhaps contrasting with the widely accepted view, our results favor a model in which CD8^+^ T_M_ cells mediate host protection during a recall infection through a targeted, “surgical” intervention. The initiating response is largely regulated by tissue-specific cues and innate immune cells before CD8^+^ T_M_ cells intervene to make the innate cellular effector response highly effective. Another unexpected finding of this work is related to IFNγ, which, at least in this context, is not sufficient to achieve full activation of CCR2^+^Ly6C^+^ monocytes and requires chemokine signaling. This result suggests that many levels of fine-tuning are involved to make a memory response most effective while preventing excessive damages to vaccinated host.

## MATERIALS AND METHODS

### Ethics statement

This study was carried out in strict accordance with the recommendations by the animal use committee at the Albert Einstein College of Medicine. All efforts were made to minimize suffering and provide humane treatment to the animals included in the study.

### Mice

All mice were bred in our specific pathogen–free animal facility at the Albert Einstein College of Medicine. We used 6- to 8-week-old WT B6 male or female mice, congenic CD45.1^+/+^ (JAX#002014), B6-K^d^ (*58*), OT-I^+^ (JAX#003831) crossed to *Rosa26*^*Actin-tomato-loxP-STOP-loxP (LSL)*–GFP^ (Td^+^) (JAX#007576), gBT-I^+^ [gift from F. Carbone (*59*)] crossed to *UBC^GFP/GFP^* (JAX#004353) or to CD45.1^+/+^ mice, *Ccr2*^DTR-CFP/WT^ [gift from E. Pamer (*60*)], *Itgax/Cd11c*^DTR/WT^ (JAX#004509), *Rosa26^CreERT2^* (JAX#008463), *Irf4^loxP/loxP (or fl/fl)^* (JAX#009380), *Ifngr1^−/−^* (JAX#003288), and *CX3CR1^ERT2Cre^* (JAX#020940) crossed to *Rosa26^LSL-PTX^* [gift from S. Coughlin (*46*)] purchased from the Jackson laboratories unless otherwise indicated. All mice are on the B6 genetic background unless otherwise specified.

### Microbial pathogens and mouse infections

#### Listeria monocytogenes

Mice were inoculated with *Lm*, *Lm* expressing the ovalbumin (*Lm*-Ova; gift from H. Shen, University of Pennsylvania) or Ova, and *HSV-2* glycoprotein B 498-505 epitope (*Lm*-Ova-gB; gift from D. Zehn, Technical University of Munich), all expressed under the LLO/*Hly* promoter. All *Lm* were prepared after passaging into WT B6 mice, by growing to log phase [optical density at 600 nm (OD_600_), ~0.3 to 0.4], and kept as frozen aliquots for single use at −80°C. For infections, bacteria were grown to a logarithmic phase (OD_600_, ~0.05 to 0.15) in broth heart infusion medium, diluted in PBS to infecting concentration, and intravenously injected. We used 0.1 × median lethal dose, i.e., 10^4^ *Lm* colony-forming units (CFUs) for primary immunizations and 10^6^ *Lm* CFUs for secondary/recall challenge infections (~6 weeks later). All *Lm* are on the 10403s genetic background.

#### Vesicular stomatitis virus

Single-use frozen aliquots of *VSV* encoding Ova (*VSV*-Ova; gift from K. Khanna, New York University) kept at −80°C were thawed and diluted in cold PBS right before mouse primary intravenous infections with 2 × 10^5^ plaque-forming units (PFUs). For secondary challenge infections of *VSV*-immunized mice (~6 weeks later), we used 10^6^ *Lm* CFUs.

### Preparation of cell suspensions for flow cytometry and adoptive transfers

Spleens were dissociated on a nylon mesh and lysed in red blood cell lysis buffer (0.83% NH_4_Cl, v/v), before incubation in Hanks’ balanced salt solution medium with collagenase I (4000 U/ml) and deoxyribonuclease I (0.1 mg/ml). BM cells were obtained by flushing femur with complete medium [RPMI 1640, 10% fetal bovine serum (FBS), 1% penicillin/streptomycin, 55 μM β-mercaptoethanol, 1 mM sodium pyruvate, 1× GlutaMAX, and 1× nonessential amino acids] containing 10% FBS.

### Cell staining for fluorescence-activated cell sorting analysis and cell sorting

Cell suspensions were incubated with 2.4G2 Fc block and stained in PBS, 1% FBS, 2 mM EDTA, and 0.02% sodium azide with fluorescently tagged Abs purchased from eBioscience, BD Biosciences, R&D Systems, or BioLegend (see details in table S3) or major histocompatibility complex class I (MHCI) (K^d^ or K^b^) Tet. For Tet, biotinylated monomers (1 mg/ml) obtained from the National Institutes of Health (NIH) Tetramer Core Facility were conjugated with PE-labeled streptavidin (1 mg/ml) as follows: 6.4 μl of PE-streptavidin was added to 10 μl of monomers every 15 min, four times on ice. Newly generated Tet were then used to stain spleen cells for 1 hour at 4°C (1:400 to 1:500 dilution). To stain for expression of the IRF4 transcription factor, cells were fixed in eBioscience Fixation/Permeabilization buffer before anti-IRF4 mAb staining in eBioscience Permeabilization buffer for 30 min. For intracellular cytokines, cells were first incubated for 4 hours at 37°C and 5% CO_2_ in complete medium, 10% FBS, with GolgiPlug/GolgiStop (brefeldin A/monensin A). Next, cells were stained for cell surface marker expression and fixed in intracellular fixation buffer (eBioscience) before permeabilization for ~1 hour in the presence of Abs/sera against intracellular cytokines (IFNγ and TNFα) and chemokines (CXCL9, CCL3, CCL4, and XCL1). For CCL3, CCL4, and XCL1, a donkey anti-goat (2 μg/ml) secondary Ab was used. Data acquisition was performed on a FACSAria III or LSRII flow cytometer. All flow cytometry data were analyzed using FlowJo v9 software (TreeStar).

Cell sorting was performed using a four-laser (405, 488, 561, and 640 nanometer) FACSAria III cell sorter from BD equipped with FACSDiva version 6.1.3. The instrument was set up with a 100-μm nozzle at 20 psi, and the samples were introduced to the system at the lowest flow rate to minimize shear stress. The sorted populations were gated to exclude double and dead cells. The sort was performed with a purity precision mode.

### Cell sorting for adoptive T cell transfers

For naïve OT-I and gBT-I cells, WT mice were adoptively transferred with ~1000 OT-I Td^+^ and 50,000 gBT-I cells isolated from the spleen of OT-I Td^+^ and gBT-I CD45.1^+/+^ mice. The next day, mice were immunized with *Lm*-Ova-gB. Immunized mice were next used ~6 weeks later to investigate OT-I and gBT-I T_M_ cell reactivation by fluorescence-activated cell sorting (FACS) and IVM. For adoptive transfers of OT-I memory cells, WT mice were first adoptively transferred with ~1000 naïve OT-I Td^+^ cells as above, immunized the next day with *VSV*-Ova, and challenged 2 weeks later with 10^6^ *Lm*-Ova. After ~4 weeks, spleens were harvested, and CD8^+^ T cells were negatively selected using anti-CD4, anti-CD11b, anti-MHCII, anti-TER119, anti-B220, and anti-CD19 mAbs (table S3), which were all added and incubated at 5 μg/ml for 30 min at 4°C. Cells were then washed and incubated with anti-rat Ab magnetic beads at one bead per target cell for 40 min at 4°C [Dynabeads sheep anti-rat immunoglobulin G (IgG), Invitrogen]. CD8^+^ T cell purity was ~70%. Cells were next sorted into 3 ml of complete media (RPMI 1640, 10% FBS, 1% penicillin/streptomycin, 55 μM β-mercaptoethanol, 1 mM sodium pyruvate, 1× GlutaMAX, and 1× nonessential amino acids) using our four-laser BD FACSAria III cell sorter. A total of 2 × 10^5^ OT-I T_M_ cells (purity, >98.5%) were transferred to either WT, *Ccr2^CFP^*, or *Ifngr1^−/−^* recipient mice further challenged with 10^6^ *Lm* or *Lm*-Ova for analysis of memory functions, protection, or IVM.

### BM transfers for CCR2^+^Ly6C^+^ monocyte chemokine receptor expression analysis

A total of 5 × 10^6^ BM cells from WT CD45.1^+/−^ and *Ifngr1^−/−^* CD45.2^+/+^ donors were cotransferred to mice immunized with *Lm*-Ova 6 weeks before and further challenged or not with *Lm*. Spleen cells were next stained for chemokine receptor expression on monocytes from BM donor-derived cells.

### Generation of BM chimera mice

Lethally irradiated 12 Gy B6-K^d^ mice were immediately reconstituted with a total of 2 × 10^6^ BM cells isolated from (i) C*cr2^DTR^*K^d^ and K^d^, (ii) C*cr2^DTR^*K^d^ and WT, (iii) C*d11c^DTR^*K^d^ and WT, and (iv) K^d^ and WT mice, at a 7:3 ratio, respectively. Donor BM cells were depleted of CD8 and CD4 T cells from WT BM cells using anti-CD8β (clone H35) and anti-CD4 (clone GK1.5) mAbs before reconstitution. Chimerism of reconstituted mice was checked ~6 weeks later in the blood, before immunizations.

### In vivo treatments

#### Monocyte and DC depletion

CD11c^+^ or CCR2^+^ cells were respectively depleted in mice expressing the DTR under the CD11c/Integrin Subunit Alpha X (Itgax) or the CCR2 promoter upon intraperitoneal injection of 10 ng/g of mouse body weight of DT (Calbiochem) 12 hours before *Lm* challenge infection.

#### Tx treatments to induce Irf4 depletion or to express PTX in CX3CR1^+^ cells

4-Hydroxytamoxifen (#T5648, Sigma-Aldrich) was dissolved in sunflower oil to a concentration of 10 mg/ml for intraperitoneal injection. For *Irf4* depletion, 3000 OT-I-*Rosa26-Cre^ERT2^Irf4^fl/fl^* and 1000 OT-I *Irf4*^WT^ were cotransferred to WT B6 mice that were next immunized with *Lm*-Ova the day after. Six weeks later and before challenge, mice were treated with Tx (1 mg per injection in 100 μl) or vehicle (100 μl of sunflower oil) for 5 days, and 24 hours after the last Tx injection, mice were challenged with *Lm*-Ova. For induction of PTX expression in CX3CR1^+^ cells, 1000 OT-I were transferred to *CX3CR1^creERT2^Rosa26^LSL-PTX^* mice that were immunized with *Lm*-Ova the next day. Six weeks later and before challenge, mice were intraperitoneally treated with Tx or vehicle as above and, the next day, challenged with *Lm*-Ova.

#### In vivo Ly6C-PE Ab labeling for CCR2^+^Ly6C^+^ monocyte staining

For IVM analysis, 10 μg of Ly6C-PE (clone HK1.4, rat IgG2a, BioLegend) mAb was inoculated to mice intravenously at the time of *Lm* challenge or 1 hour before euthanizing mice. For FACS analysis, 5 μg of Ly6C-PE mAb was injected to challenged mice 1 hour before the euthanasia.

### In vitro activation assays

#### Quantification of CCL3 and IFNγ secretion

A total of 10^6^ splenocytes from mice immunized with *Lm*-Ova and challenged or not 6 weeks later with *Lm*-Ova for 16 hours were incubated in 96-well flat-bottom plate with complete medium only or in the presence of Golgi Plug/Stop for 4 hours at 37°C. CCL3 (Thermo Fisher Scientific) and IFNγ (BioLegend) production in culture supernatants was then quantified by enzyme-linked immunosorbent assay.

#### Measure of chemokine expression by ex vivo restimulated OT-I T_M_ cells

Splenocytes from mice immunized with *Lm-*Ova 6 weeks prior were coincubated with SIINFEKL peptide (10^−8^ M) with Golgi Plug/Stop and (i) with or without cycloheximide (translation inhibitor; 10 μg/ml; Sigma-Aldrich) or actinomycin D (transcription inhibitor; 8 μM; Sigma-Aldrich) for 1, 2, 3, and 4 hours in complete medium at 37°C and (ii) with the SCG-CBP30 IRF4 inhibitor (20 μM; Selleckchem). Cells were next stained as described above including for intracellular expression of CCL3 and/or CCL4 and XCL1.

#### *Measure of TNF*α *expression in ex vivo stimulated CCR2^+^Ly6C^+^ monocytes and neutrophils*

A total of 10^4^ monocytes or neutrophils were FACS-sorted (as above; purity, >98%) from mice primary immunized and challenged with *Lm*-Ova 6 weeks later for 16 hours. Cells were next coincubated in 96-well round-bottom plate and complete medium, with HK*Lm*, rCCL3, rCCL4, rXCL1, or the combination of the three recombinant chemokines, in the presence of GolgiPlug/Stop for 4 hours at 37°C before staining for cell surface and intracellular markers for FACS analysis. In CCR5 and CCR1 blocking experiments, cells were incubated with both CCR5 (1 μM; Maraviroc, Cayman Chemical Company) and CCR1 (1 μM; J113863, Santa Cruz Biotechnology) chemical inhibitors or control dimethyl sulfoxide 30 min before adding recombinant chemokines or HK*Lm*.

#### *Measure of TNF*α *and CXCL9 expression in in vitro cocultured CCR2^+^Ly6C^+^ monocytes and OT-I T_M_ cells*

A total of 8 × 10^4^ OT-I T_M_ cells were FACS-sorted (as above; purity, >98%) and stimulated with SIINFEKL peptide (10^−6^ M) OVN in complete media (37°C, CO_2_). The next day, we sorted 2 × 10^4^ CCR2^+^Ly6C^+^ monocytes from a naïve spleen based on the cell surface expression of CD11b and Ly6C and CD8 exclusion. Sorted monocytes (purity, >98.5%) were next added in the presence of Golgi Plug/Stop to the T cells with or without anti-CCL3, CCL4, and XCL1 chemokine-neutralizing antisera (table S3). As control, naïve sorted monocytes were also incubated with the three recombinant chemokines rCCL3, rCCL4, and rXCL1 (table S3) before staining for cell surface and intracellular markers for FACS analysis.

### RNA sequencing

#### Samples and library preparation

A total of 1000 OT-I Td^+^ and 1000 gBT-I CD45.1^+/+^ T_M_ cells were flow-purified (FACSAria III) following the same procedure as for adoptive T_M_ cell transfers described before, from 6-week *Lm*-Ova-gB–immunized mice either left unchallenged or challenged with *Lm*-Ova. However, here, T_M_ cells were directly sorted into 1× lysis buffer, and complementary DNA (cDNA) was synthesized and amplified directly from intact cells using the SMART-Seq v4 Ultra Low Input RNA Kit for sequencing (Takara Bio, USA) according to the manufacturer’s protocol. cDNA was isolated using the Agencourt AMPure XP Kit (Beckman Coulter, Brea, CA) and quantified using the Qubit dsDNA High Sensitivity Assay Kit (Life Invitrogen) on an Agilent 2100 Bioanalyzer (Agilent Technologies, Santa Clara, CA). The library preparation was performed using the Nextera XT DNA Library Preparation Kit (Illumina Inc., San Diego, CA). Samples were sequenced to depths of up to 16.7 million single-end 75-nucleotide length reads per sample using the Illumina NextSeq 500/550 High Output v2 kit (75 cycles) on an Illumina NextSeq 500 Sequencing System. Image analysis, base calling, and generation of sequence reads were produced using the NextSeq Control software v2.0 and Real-Time Analysis software v2. Data were converted to FASTQ files using the bcl2fastq2 v2.20 software (Illumina Inc.). Sequencing data were initially quality-checked using FastQC, before alignment and initial analysis. Reads were aligned to the mouse reference mm10 using STAR aligner (v2.4.2a) (*61*). Quantification of genes annotated in Gencode vM5 was performed using featureCounts (v1.4.3), and quantification of transcripts was performed using Kalisto (*62*). Quality check was collected with Picard (v1.83) and RSeQC (*63*) (http://broadinstitute.github.io/picard/). Normalization of feature counts was performed using the DESeq2 package version 1.10.1. Before analysis, nonrelevant batch effect (such as library preparation or sequencing batch) was identified using unsupervised PCA, and analysis was corrected for batch effects through our model. Differentially expressed genes were identified using negative binomial distribution as implemented in DESeq 2 [R package (*64*)]. Significantly up-regulated and down-regulated genes (differentially expressed gene) were defined with a false discovery rate step-up *P* ≤ 0.05 and a fold change of ≥±1.5. The raw data from the National Center for Biotechnology Information database (GEO GSE160280) were subsequently analyzed for enrichment of GO terms and the Kyoto Encyclopedia of Genes and Genomes (KEGG) pathways, implemented in the clusterProfiler (R package, function enrichGO or enrichKEGG); a pathway is considered significantly enriched if the enrichment score is ≥1.5 (equivalent to *P* ≤ 0.05).

### Intravital and explant imaging

For intravital imaging, mice were anesthetized with isoflurane, and the spleen was surgically exposed and elevated above the body of the mouse. A glass coverslip was carefully applied to the top of the spleen to create an imaging window. Mice were kept at 37°C using a custom heating platform. Imaging was performed on an Olympus FVE-1200 upright microscope using a 25× 1.04–numerical aperture objective and a Deepsee MaiTai Ti-Sapphire pulsed laser (Spectra Physics) tuned to 870 nm. To maintain temperature and limit infiltrating light, the microscope was fitted with a custom-built incubator chamber heated to 37°C. Z-stack images (512 by 512) were acquired every 60 s with 5-μm steps. For explant imaging, mice were euthanized with CO_2_, and spleens were immediately harvested. Spleens were affixed to coverglass using Vetbond (3M) on the medulla. Tiled images were acquired using 320 × 320 Z-stack images with 15-μm steps. Tiled images were stitched using Olympus Fluoview software. Cell tracking, drift correction, and monocyte volume analysis were carried out using Imaris 9.2 (Bitplane).

### Measure of protective immunity

Protection was measured by enumerating *Lm* titers in spleens and livers of challenged mice. For this, spleens and livers were harvested 24 hours after infection and dissociated on metal screens in 10 ml of water/0.1% Triton X-100 (Sigma-Aldrich). Serial dilutions were performed in the same buffer, and 100 μl was plated onto brain heart infusion (BHI) medium plates. *Lm* CFU numbers were counted 24 hours later and reported to the whole spleen and liver. This procedure was the same whether mice were (i) primary challenged and transferred or not with OT-I memory cells, (ii) primary/boosted or not, and secondary challenged treated or not with Tx (*CX3CR1^creERT2^Rosa26^LSL-PTX^*, OT-I *Rosa26-Cre^ERT2^ Irf4^fl/fl^*).

### Statistics

Statistical significance was calculated using an unpaired Student’s *t* test with GraphPad Prism software, and two-tailed *P* values are given as follows: *P < 0.1, **P < 0.01, ***P < 0.001, ****P < 0.0001, and not significant (ns) for *P* > 0.1. All *P* values of 0.05 or less were considered significant.

## Notes

View/request a protocol for this paper from *Bio-protocol*.
