# Supplementary material for: Memory CD8+ T cells mediate early pathogen-specific protection via localized delivery of chemokines and IFNγ to clusters of monocytes
Source: Sci Adv. 2021 Sep 1;7(36):eabf9975. doi: 10.1126/sciadv.abf9975 (PMC8442869; doi:10.1126/sciadv.abf9975)
Supplement: Supplementary file 1 — Figs. S1 to S8 Legends for tables S1 to S3 Legends for movies S1 to S3 [file sciadv.abf9975_sm.pdf]

Science Advances
AAAS

Supplementary Materials for

Memory CD8$^+$ T cells mediate early pathogen-specific protection via localized delivery of chemokines and IFNγ to clusters of monocytes

Marie Boutet, Zachary Benet, Erik Guillen, Caroline Koch, Saidi M'Homa Soudja, Fabien Delahaye, David Fooksman*, Grégoire Lauvau*

*Corresponding author. Email: gregoire.lauvau@einsteinmed.org (G.L.); david.fooksman@einsteinmed.org (D.F.)

Published 1 September 2021, *Sci. Adv.* **7**, eabf9975 (2021)
DOI: 10.1126/sciadv.abf9975

The PDF file includes:

Figs. S1 to S8
Legends for tables S1 to S3
Legends for movies S1 to S3

Other Supplementary Material for this manuscript includes the following:

Tables S1 to S3
Movies S1 to S3

Supplemental Figure Legends:

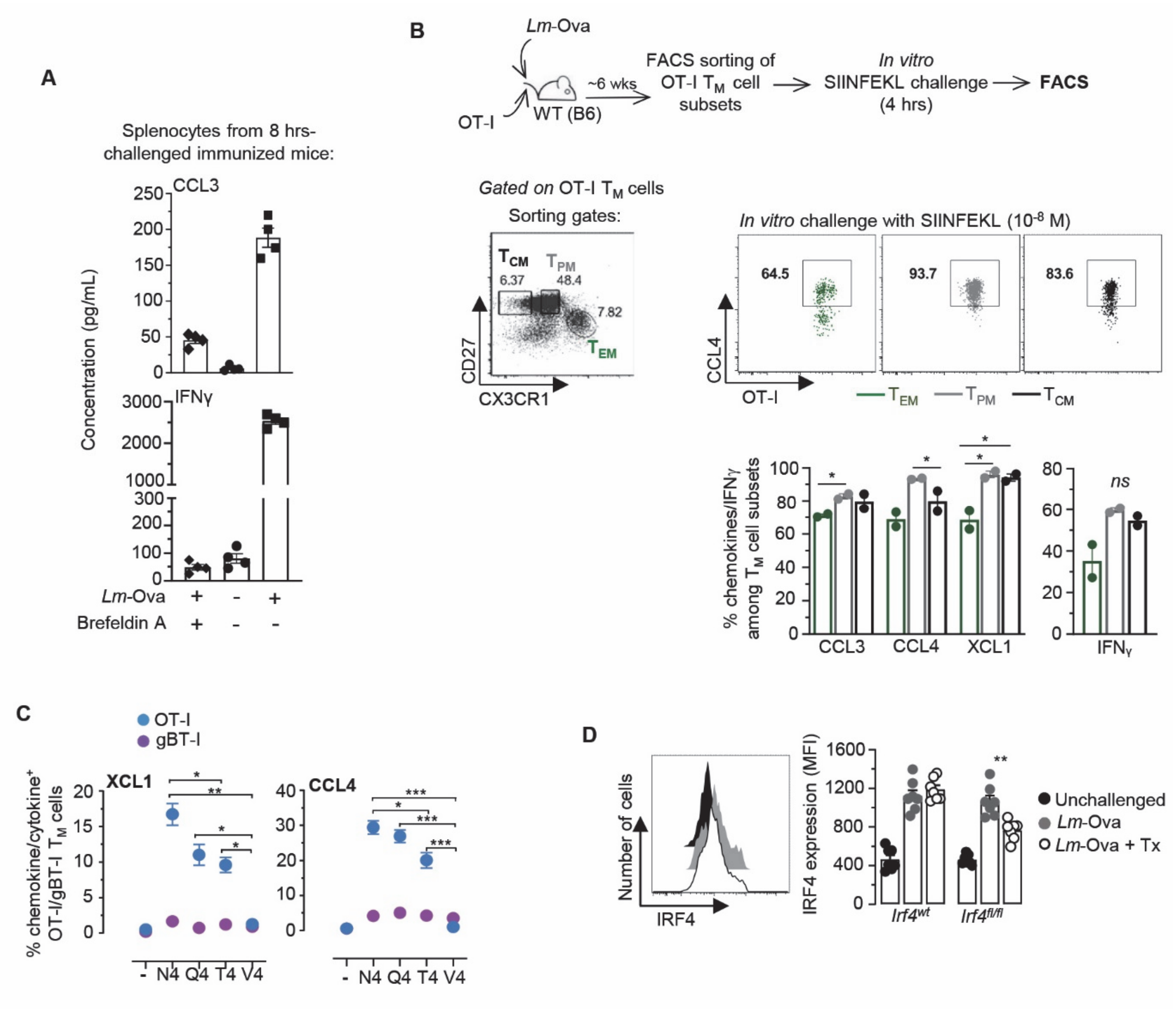

Figure S1, related to Figures 2 and 3. (A) Spleen cells were isolated from *Lm*-Ova-immunized mice (10^4) that were rechallenged 6 wks later with 10^6 *Lm*-Ova. At 8 hrs post challenge, spleen cells were incubated or not with Golgi plug/stop, before collecting supernatants 4 hrs later. CCL3 and IFNγ were next quantified by ELISA. (B) Subsets of OT-I T_M cells were flow-sorted from the spleens of mice transferred with naïve OT-I cells and immunized with 10^4 *Lm*-Ova 6 wks before (schematic), based on CX3CR1 and CD27 cell surface marker expression (T_{EM}: CX3CR1^{hi}CD27^{low}, T_{PM}: CX3CR1^{int}CD27^{hi}, T_{CM}: CX3CR1^{low}CD27^{hi}). Sorted (gates are shown) OT-I T_M subsets were next stimulated for 4 hrs with the SIINFEKL peptide (10^{-8}M) *in vitro* and stained for cell-surface CD8, CXCR3, KLRG1 and intracellular CCL3, CCL4, XCL1 and IFN$_γ$. Representative dot plots with summary bar graphs (each symbol is 1 mouse) show the proportion of chemokines$^+$ or cytokines$^+$ cells among CD8$^+$ T_M subsets with indicated p-value. (C) Mice grafted with OT-I Td$^+$ and CD45.1$^{+/+}$ gBT-I cells were immunized with 10^4 *Lm*-Ova-gB, and ~6 wks later challenged or not for 16 hrs with 10^6 *Lm*-Ova N4 or *Lm* expressing 3 different Ova APLs, namely *Lm*-Ova Q4, *Lm*-Ova T4 or *Lm*-Ova V4. Spleen cell suspensions were next incubated with Golgi Plug/Stop and stained for cell-surface CD8, CD3, CD45.1 and indicated intracellular XCL1 or CCL4 chemokines. Graphs represent the proportion of OT-I or gBT-I T_M cells expressing indicated intracellular markers after challenge with *Lm*-expressing N4, Q4, T4 or V4. (D) *Rosa26*^{CreERT2}*Irf4*$^{flox/flox}$*Cd45.2*$^{+/+}$ and *Irf4*^{wt} *Cd45.1*$^{+/-}$ OT-I cells were co-transferred to *Cd45.1*$^{+/+}$ WT recipient mice and immunized with 10^4 *Lm*-Ova the next day. Six wks later, mice received Tx (1mg/day) or vehicle i.p. every day for 5 days before secondary challenge infection

with 10^6 *Lm*-Ova. At 16 hrs, IRF4 expression was determined as described above in *Irf4*$^{flox/flox}$ versus *Irf4*^{wt} OT-I T_M cells in the different experimental conditions. Representative FACS dot plots and histograms staining are shown. Panels pool the result of 2 independent replicate experiments with n=6 (C) and 7 (D) mice. P-values are indicated.

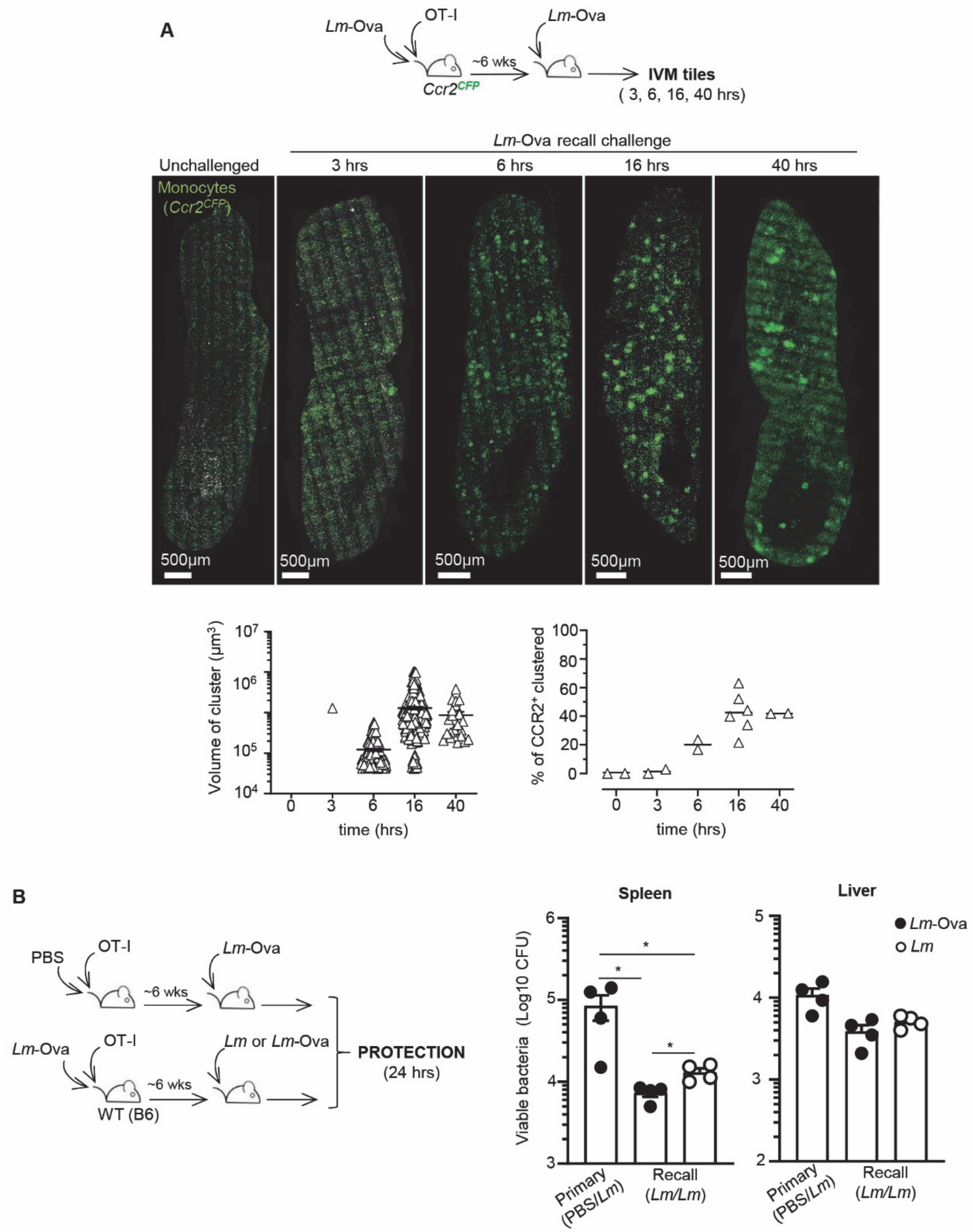

Figure S2, related to Figure 4. (A) *Ccr2*^{CFP} mice grafted with OT-I cells were immunized with 10^4 *Lm*-Ova and 6 wks later challenged or not with 10^6 *Lm*-Ova for 3, 6, 16 and 40 hrs. Ccr2^{CFP} monocytes are in green and representative mouse spleen tile reconstructions are shown. Graphs shows the volume of individual clusters and the average proportion of CCR2$^+$Ly6C$^+$ monocytes clustered in a pool of 2 independent experiments (n=2-5 mice). (B) Age- and sex-matched WT B6

mice were transferred with OT-I cells and injected with PBS or immunized with 10^4 *Lm*-Ova. 6 wks later, PBS-injected mice were challenged with 10^6 *Lm*-Ova ("Primary"), and *Lm*-Ova-immunized mice were challenged with 10^6 *Lm* or *Lm*-Ova ("Recall"). Bacterial titers in spleens and livers were determined 24 hrs post challenge. Bar graphs represent 1 of 2 representative experiments with each symbol corresponding to an individual mouse (n=4 mice).

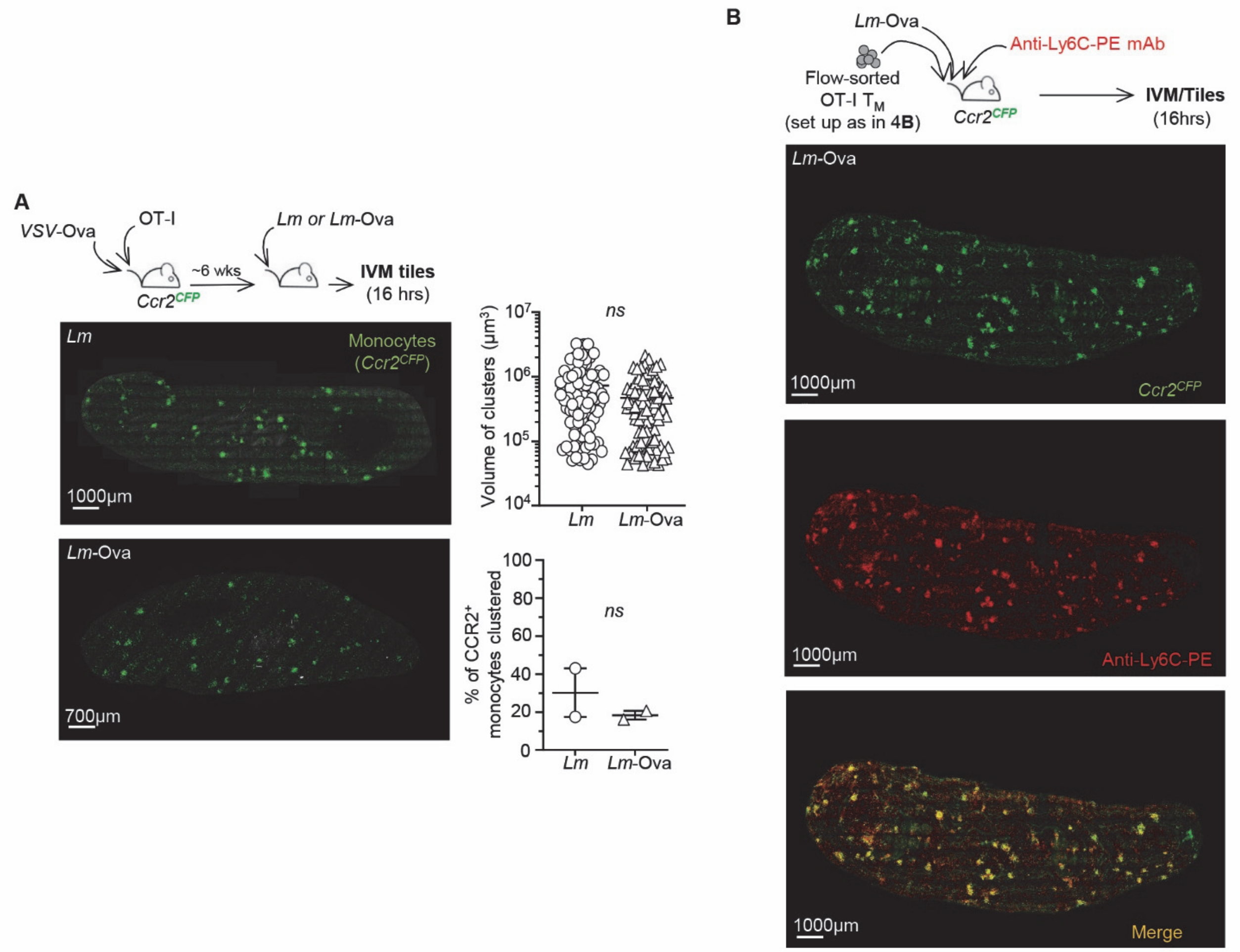

Figure S3, related to Figure 4. (A) *Ccr2*^{CFP} mice grafted with OT-I cells were immunized with *VSV*-Ova (2x10^5 PFU) and 6 wks later challenged with 10^6 *Lm* or *Lm*-Ova for 16 hrs. Ccr2^{CFP} monocytes are in green and representative tiles of reconstructed mouse spleens are shown. Graphs show the volume of individual clusters and the average proportion of CCR2$^+$Ly6C$^+$ monocytes clustered in 2 independent replicate experiments (n=2 mice). (B) 2x10^5 flow-sorted- OT-I T_M cells generated as depicted in Figure 4B, were transferred to naive *Ccr2*^{CFP} that were also injected with Ly6C-PE mAb (10µg) and 10^6 *Lm*-Ova for 16 hrs. Representative tiles of reconstructed mouse spleens in 1 of 2 replicate experiments are shown with Ccr2^{CFP} monocytes in green and Ly6C-PE$^+$ monocytes in red (n=4 mice). Green and red signals are merged in yellow.

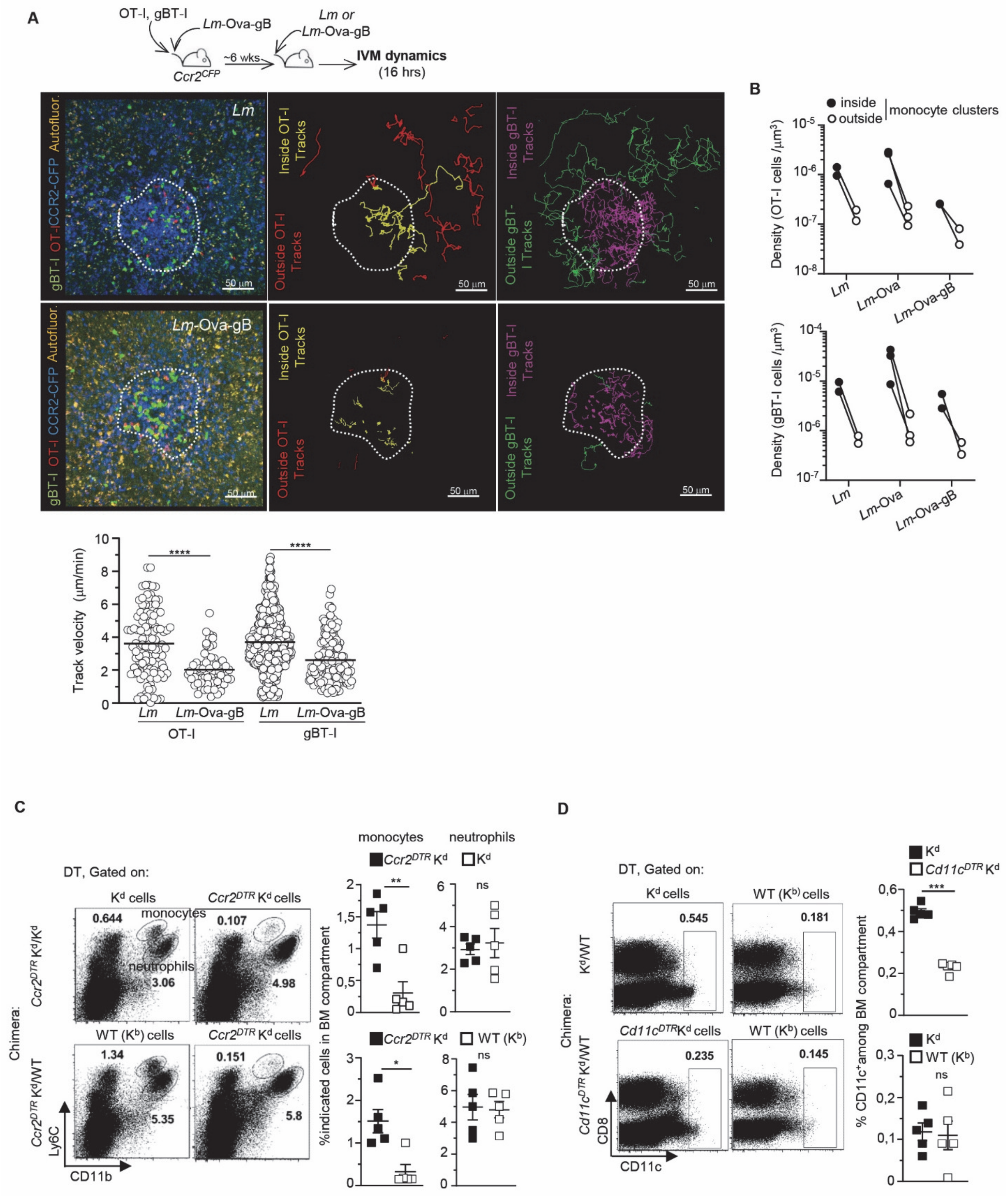

Figure S4, related to Figure 5. *Ccr2^{CFP}* mice were co-transferred with naïve OT-I Td^{+} and gBT-I GFP^{+} cells and immunized with 10^4 *Lm*-Ova-gB. Six wks later, mice were challenged with 10^6 *Lm* or *Lm*-Ova-gB and IVM images in the spleen of live mice were recorded 16 hrs later. (A) Representative IVM image (right) of OT-I (red) and gBT-I (green) T$_M$ cells localized in a cluster (delimited area by white dashed line) of CCR2^{CFP} monocytes (blue) are shown with autofluorescence (yellow). OT-I T$_M$ (outside, red and inside, yellow) and gBT-I T$_M$ cell tracks (outside, green and inside, purple) inside and outside the cluster of CCR2^{CFP} monocytes are also shown (center and left images). Graphs represent the speeds of OT-I and gBT-I T$_M$ cells in the

clusters. (B) Graphs represent the density of OT-I or gBT-I T_M cells inside and outside of monocyte clusters after 10^6 *Lm, Lm*-Ova or *Lm*-Ova-gB challenges. (C, D) Efficiency of DT-mediated depletion in indicated groups and compartments of mixed BM chimeras. Representative FACS dot plots in a pool of 2 experiments with p-value are shown (n=5 mice).

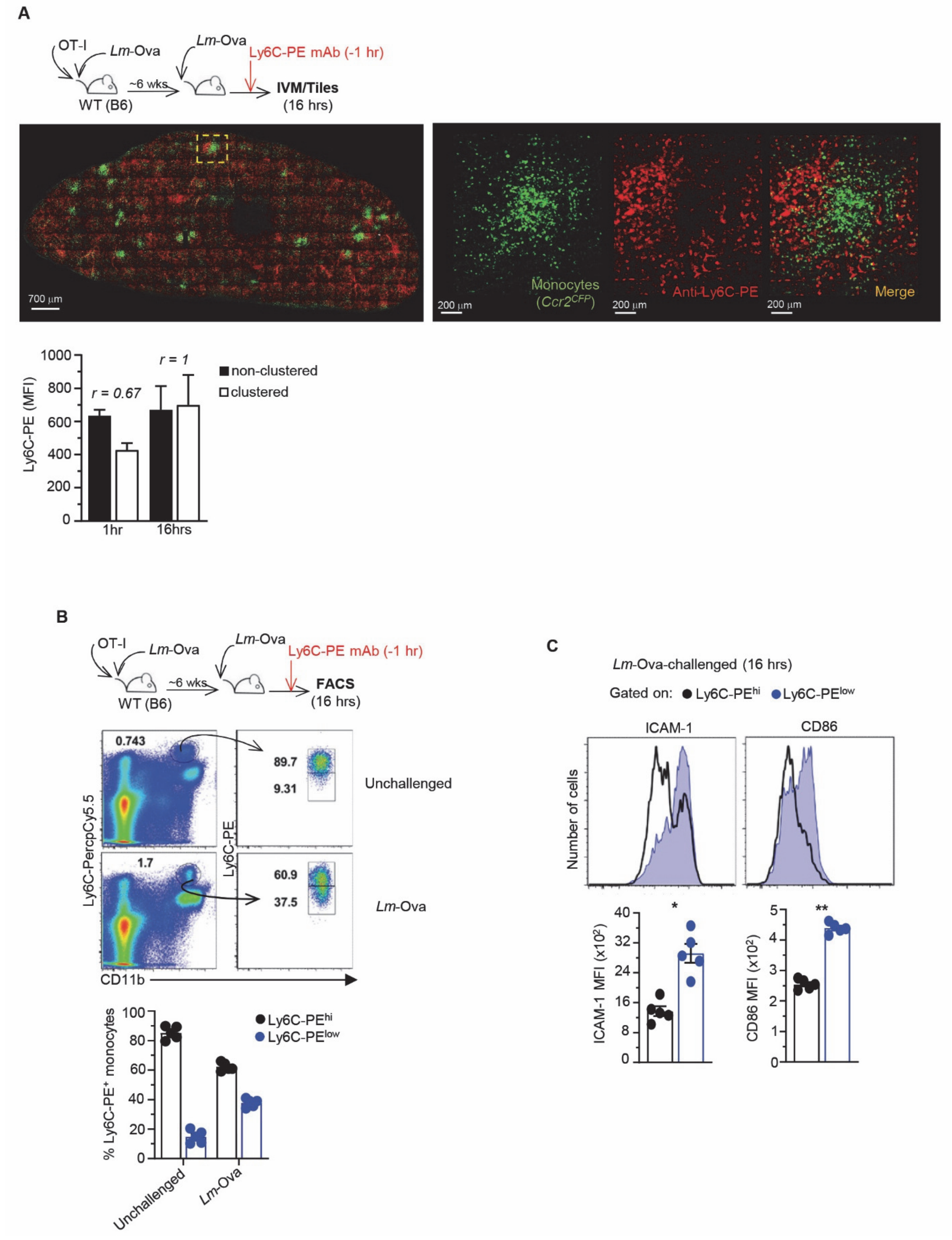

Figure S5, related to Figure 6. (A-B) *Lm*-Ova-immunized mice were co-transferred with naive OT-I Td^{+} cells and challenged or not ~6 wks later with *Lm*-Ova for 16 hrs. 1 hr before sacrifice, mice were injected i.v. with Ly6C-PE mAb. (A) Representative tiles of reconstructed mouse spleens in 1 of 2 replicate experiments are shown with Ccr2^{CFP} monocytes in green and Ly6C-PE^{+} monocytes in red. Green and red signals are merged in yellow. Bar graphs represent the intensity of Ly6C-PE staining (MFI) on non-clustered and clustered CCR2^{+}Ly6C^{+} monocytes at 1 or 16 hrs

post Ly6C-PE mAb injection across 2 replicate experiments (n=2-4 mice). *r* corresponds to the ratio of MFI between clustered and non-clustered $CCR2^{+}Ly6C^{+}$ monocytes. (B) Gating strategy to identify by flow cytometry $Ly6C\text{-}PE^{hi}$ and $Ly6C\text{-}PE^{low}$ monocytes, after gating on Ly6C-$PerCpCy5.5^{+}$ monocytes following the experimental design as described above. (C) Representative dot plots and FACS histograms of cell-surface ICAM-1 and CD86 expression on $Ly6C\text{-}PE^{hi}$ and $Ly6C\text{-}PE^{low}$ monocytes are shown. Bar graphs pool 2 independent replicate experiments with each symbol corresponding to one mouse and indicated p-value (n=5 mice).

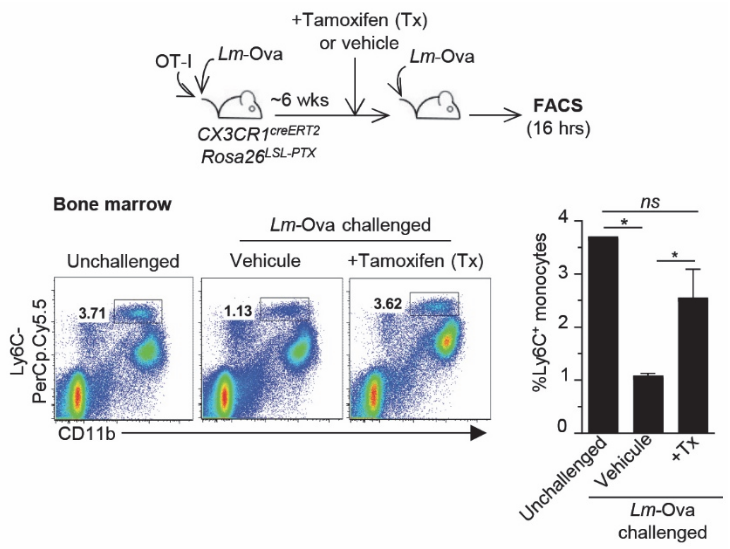

Figure S6, related to Figure 7. *CX3CR1*^{CreERT2}*Rosa26*$^{LSL\text{-}PTX}$ mice transferred with OT-I cells were immunized with 10^{4} *Lm*-Ova. 6 wks later, mice received Tx or vehicle i.p. daily for 5 days prior to 10^{6} *Lm*-Ova recall infection. 16 hrs post challenge infection, BM cells (femur) were isolated and stained for cell surface CD11b, Ly6C-PerCpCy5.5 and the proportion of $CCR2^{+}Ly6C^{+}$ monocytes were quantified. Representative FACS dot plots are shown and bar graphs pool 2 independent replicate experiments with n=6. A naive group of aged-matched mice ("unchallenged") were included as control of BM monocyte proportions. Bar graphs show 1 of 2 independent experiments with n=3-4 mice. P-values are indicated.

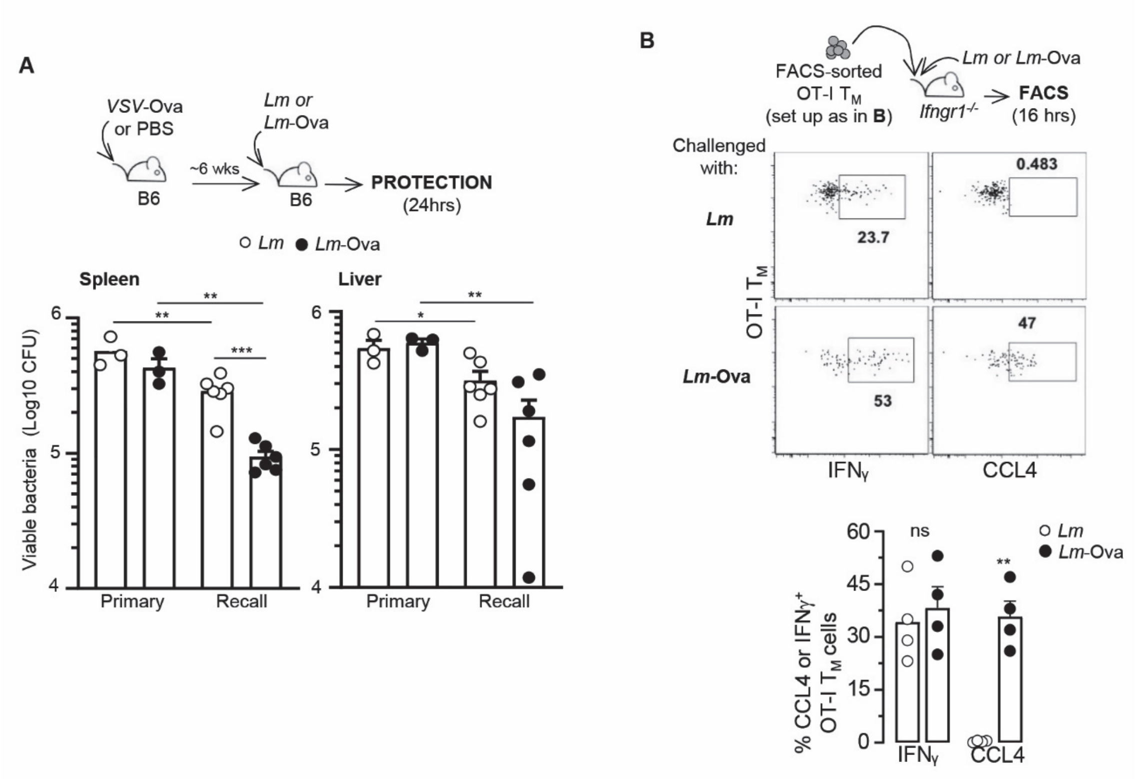

Figure S7, related to Figure 8. (A) Mice were immunized with $2x10^5$ PFU *VSV*-Ova or injected with PBS, and ~6 wks later challenged with 10^6 *Lm* or *Lm*-Ova. Spleens and livers from challenged mice were harvested 24 hrs later and *Lm* CFUs determined after plating. Bar graphs show 1 of 2 representative experiments with each symbol corresponding to 1 individual mouse. (B) $2x10^5$ OT-I T_M cells induced using the depicted experimental set up, were transferred in *Ifngr1*$^{-/-}$ mice, and mice were next challenged with 10^6 *Lm* or *Lm*-Ova for 16 hrs. OT-I Td^+ T_M cells were stained for cell surface CD8, CD3 and intracellular CCL4 and IFNγ. Representative FACS dot plots are shown and bar graphs pool 2 independent replicate experiments (n=4 mice) with indicated p-values.

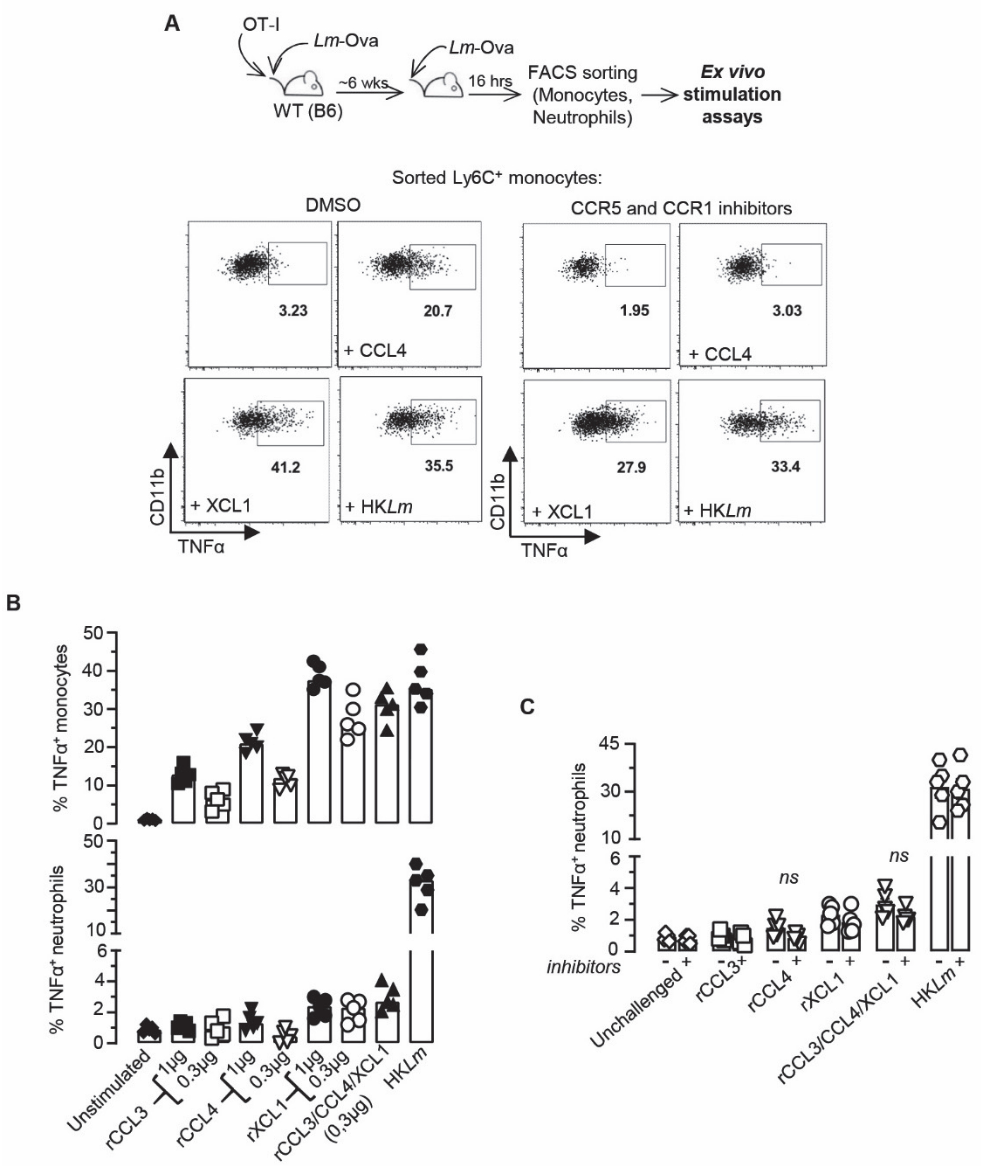

Figure S8, related to Figure 9. (A, B) Mice grafted with OT-I cells were immunized with 10^4 *Lm*-Ova and 6 wks later challenged with 10^6 *Lm*-Ova. 16 hrs post-challenge, $CCR2^+Ly6C^+$ monocytes and neutrophils were sorted from spleen and stimulated for 4 hrs with or without recombinant chemokines at indicated concentrations, or with HK*Lm* and with or without CCR1/5 inhibitors (as depicted in Figure 9B). Cells were next stained for cell surface expression of CD11b, Ly6C, Ly6G and intracellular TNFα. Representative FACS dot plots are shown. (B) Graphs show $TNF\alpha^+$ monocytes and neutrophils frequency after 4 hrs incubation with recombinant chemokines (1 and 0.3µg) or HK*Lm*. Bar graphs (each symbol is 1 mouse) represent the pool of 2 independent replicate experiments with p-values indicated. (C) TNFα expression in FACS-sorted neutrophils as depicted in Figure 6C, incubated with CCR5 and CCR1 inhibitors or DMSO vehicle. Bar graphs (each symbol is 1 mouse) represent the pool of 2 independent replicate experiments with p-values indicated.

Movie S1. Dynamic behavior of cognate antigen- *versus* inflammation-stimulated memory $CD8^+$ T cells in $CCR2^+Ly6C^+$ monocyte clusters during recall infection. Representative time-lapse movie showing cognate antigen (OT-I, red) and inflammation-stimulated gBT-I (green) $CD8^+$ T_M cells in $CCR2^+Ly6C^+$ monocyte clusters ($CCR2^{CFP}$, blue) at ~16 hrs post challenge with *Lm*-Ova.

Movie S2. Dynamic behavior of inflammation-stimulated memory $CD8^+$ T cells in $CCR2^+Ly6C^+$ monocyte clusters during recall infection. Representative time-lapse movie showing inflammation-stimulated (OT-I, red and gBT-I, green) $CD8^+$ T_M cells in $CCR2^+Ly6C^+$ monocyte clusters ($CCR2^{CFP}$, blue) at ~16 hrs post challenge with *Lm*.

Movie S3. Dynamic behavior of cognate antigen-stimulated memory $CD8^+$ T cells in $CCR2^+Ly6C^+$ monocyte clusters during recall infection. Representative time-lapse movie showing cognate antigen (OT-I, red and gBT-I, green) $CD8^+$ T_M cells in $CCR2^+Ly6C^+$ monocyte clusters ($CCR2^{CFP}$, blue) at ~16 hrs post challenge with *Lm*-Ova-gB.

Table S1. List of genes up/down regulated for Ag/Infl., Infl. and Ag/Inf./Infl $CD8^+$ T_M cells as defined in Figure 1.

Table S2. GO pathways for Figure 1E

Table S3. Table for antibodies and other reagents
